# Systematic Review and Meta‐Analysis of Medication Reviews Conducted by Pharmacists on Cardiovascular Diseases Risk Factors in Ambulatory Care

**DOI:** 10.1161/JAHA.119.013627

**Published:** 2019-11-12

**Authors:** Francisco Martínez‐Mardones, Fernando Fernandez‐Llimos, Shalom I. Benrimoj, Antonio Ahumada‐Canale, José Cristian Plaza‐Plaza, Fernanda S. Tonin, Victoria Garcia‐Cardenas

**Affiliations:** ^1^ Graduate School of Health University of Technology Sydney Australia; ^2^ Institute for Medicines Research (iMed.ULisboa) Department of Social Pharmacy Faculty of Pharmacy University of Lisbon Portugal; ^3^ Member of the Pharmaceutical Care Research Group University of Granada Faculty of Pharmacy Campus Universitario Cartuja Granada Spain; ^4^ Faculty of Chemistry and Pharmacy Pontifical Catholic University of Chile Santiago Chile; ^5^ Pharmaceutical Sciences Postgraduate Programme Federal University of Paraná Curitiba Brazil

**Keywords:** cardiovascular risk factors, hypertension, medication reviews, pharmacist management, type 2 diabetes mellitus, Hypertension, Health Services, Meta Analysis, Cardiovascular Disease, Diabetes, Type 2

## Abstract

**Background:**

Pharmacists‐led medication reviews (MRs) are claimed to be effective for the control of cardiovascular diseases; however, the evidence in the literature is conflicting. The main objective of this meta‐analysis was to analyze the impact of pharmacist‐led MRs on cardiovascular disease risk factors overall and in different ambulatory settings while exploring the effects of different components of MRs.

**Methods and Results:**

Searches were conducted in PubMed, Web of Science, Embase, the Cumulative Index to Nursing and Allied Health Literature, and the Cochrane Library Central Register of Controlled Trials database. Randomized and cluster randomized controlled trials of pharmacist‐led MRs compared with usual care were included. Settings were community pharmacies and ambulatory clinics. The classification used for MRs was the Pharmaceutical Care Network Europe as basic (type 1), intermediate (type 2), and advanced (type 3). Meta‐analyses in therapeutic goals used odds ratios to standardize the effect of each study, and for continuous data (eg, systolic blood pressure) raw differences were calculated using baseline and final values, with 95% CIs. Prediction intervals were calculated to account for heterogeneity. Sensitivity analyses were conducted to test the robustness of results. Meta‐analyses included 69 studies with a total of 11 644 patients. Sample demographic characteristics were similar between studies. MRs increased control of hypertension (odds ratio, 2.73; 95% prediction interval, 1.05–7.08), type 2 diabetes mellitus (odds ratio, 3.11; 95% prediction interval, 1.17–5.88), and high cholesterol (odds ratio, 1.91; 95% prediction interval, 1.05–3.46). In ambulatory clinics, MRs produced significant effects in control of diabetes mellitus and cholesterol. For community pharmacies, systolic blood pressure and low‐density lipoprotein values decreased significantly. Advanced MRs had larger effects than intermediate MRs in diabetes mellitus and dyslipidemia outcomes. Most intervention components had no significant effect on clinical outcomes and were often poorly described. CIs were significant in all analyses but prediction intervals were not in continuous clinical outcomes, with high heterogeneity present.

**Conclusions:**

Intermediate and advanced MRs provided by pharmacists may improve control of blood pressure, cholesterol, and type 2 diabetes mellitus, as statistically significant prediction intervals were found. However, most continuous clinical outcomes failed to achieve statistical significance, with high heterogeneity present, although positive trends and effect sizes were found. Studies should use a standardized method for MRs to diminish sources of these heterogeneities.

**Clinical Perspective**

**What Is New?**

Pharmacist‐led medication reviews (MRs) seem to improve the control of hypertension, type 2 diabetes mellitus, and dyslipidemias in ambulatory settings despite differences in components implemented and high heterogeneity between studies.
MRs in ambulatory clinics could have larger effects in the achievement of type 2 diabetes mellitus and dyslipidemia goals and in decreasing systolic blood pressure and low‐density lipoprotein cholesterol in community pharmacies.
Advanced MRs could have larger effects than intermediate MRs on diastolic blood pressure, glycated hemoglobin, fasting glucose, total cholesterol, and low‐ and high‐density lipoprotein cholesterol, but more studies are needed.

**What Are the Clinical Implications?**

- Including pharmacists in care teams to provide MRs in both community pharmacies and ambulatory clinics could improve the management of hypertension, type 2 diabetes mellitus, and dyslipidemias.

## Introduction

Cardiovascular diseases (CVDs) are the main cause of morbidity and mortality worldwide, with more than 36% of adults in the United States and 40% in Europe at high risk for developing or with established CVD.^1^, ^2^ The World Health Organization reported 17.9 million of CVD‐related deaths in 2016, representing 44% of all deaths from noncommunicable diseases, with 85% of these deaths caused by strokes and ischemic heart diseases.^3^, ^4^ Dyslipidemia, hypertension, and type 2 diabetes mellitus (T2DM) are the most common risk factors in adults, with an estimated 39%, 31%, and 8% affected worldwide, with great impact in mortality, morbidity, and costs of care. However, common strategies to control these diseases appear to be relatively ineffective.^2^, ^3^, ^4^

Pharmacists are increasingly having direct involvement in patient care usually by providing services that have the objective of improving medication management of patients and other healthcare professionals.^5^, ^6^, ^7^, ^8^ There are various types of services, including medication reviews (MRs).^8^, ^9^ MRs vary from a brief revision of the prescribed medicines to more complex interventions involving patients and physicians, which allow the detection of pharmacological interactions and drug‐related problems such as adverse drug reactions, effectiveness problems, nonadherence, and self‐medication.^10^, ^11^ Pharmacists‐led interventions have reportedly increased the achievement of therapeutic goals in CVD risk factors such as hypertension and T2DM, decreasing systolic blood pressure (BP) between 6 and 10 mm Hg and glycated hemoglobin (HbA_1c_) between 0.46% and 1%.^6^, ^7^, ^8^, ^9^, ^10^

Some systematic reviews and meta‐analyses reveal high inconsistencies and heterogeneity on the impact of MR. Possible causes of this problem are the lack of control of confounding factors such as age and other demographic data, months of follow‐up, control groups without usual care or dummy interventions, variability, and fidelity of the intervention including different settings.^5^, ^6^, ^7^, ^8^, ^9^, ^10^ These specific setting elements could include access to care teams for proposed action plans, proximity and relationship with prescribers, the physical place of the intervention, and other related factors.^6^, ^7^, ^8^, ^9^, ^10^ How these differences in ambulatory settings could influence the clinical impact of the pharmacist's provision of MR has not been reported.

The main objective of this meta‐analysis was to analyze the impact of pharmacist‐led MRs on CVD risk factors overall and in different ambulatory settings while exploring the effects of different components of MRs.

## Methods

### Data Sources and Searches

A systematic review was performed using the PRISMA statement and Cochrane Collaboration recommendations.^12^, ^13^, ^14^ Two reviewers (F.M.‐M., A.A.‐C.) performed all of the steps individually, and any discrepancies were decided by a third author (V.G.‐C.). Searches were conducted in PubMed, Web of Science, Embase (through Ovid), the Cumulative Index to Nursing and Allied Health Literature, and the Cochrane Library Central Register of Controlled Trials database, without any time limit (up to May 2019). A manual search in the reference lists of included studies was performed, and grey literature (eg, Google) was also searched. The complete search strategy for each database is available in Table S1.

### Eligibility Criteria

Table 1 describes inclusion and exclusion criteria. The Pharmaceutical Care Network Europe (PCNE) categories of MR conducted by pharmacists were used to classify interventions as^11^: type 1: a basic review of medicines and health problems based on the available medication history in the pharmacy; type 2: an intermediate review with the available medication history in the pharmacy and clinical records or information obtained directly from the patient; and type 3: an advanced review using medication history, clinical records, and information obtained directly from the patient.

**Table 1:** Inclusion and Exclusion Criteria

Category	Inclusion Criteria
Population	Patients older than 18 years with hypertension, T2DM, or dyslipidemia as CVD risk factors
Setting	Ambulatory care settings as ACs or CPs
Study design	RCT or cluster RCT
Intervention	Medication reviews provided by pharmacists describing the components of the intervention
Comparator	Usual care
Outcomes	Studies that include at least 1 of the outcomes of study. Outcomes were dichotomic as the control of hypertension; T2DM and dyslipidemia as achievement of clinical targets defined in each study; and continuous as systolic blood pressure, diastolic blood pressure, glycated hemoglobin, fasting glucose, total cholesterol, low‐density lipoprotein cholesterol, high‐density lipoprotein cholesterol, and triglycerides
Language of publication	English or Spanish
Category	Exclusion criteria
Missing data	Studies that report incomplete values (as lacking uncertainty) when the authors could not provide this information when requested

ACs indicates ambulatory clinics; CPs, community pharmacies; CVD, cardiovascular disease; RCT, randomized controlled trial; T2DM, type 2 diabetes mellitus.

During the screening phase (title and abstract reading), articles were excluded if considered irrelevant to the study goals. The full‐text eligibility phase excluded articles that did not fulfill all of the inclusion criteria.

### Data Extraction

Standardized data collection forms were used to extract data on the studies’ metadata (eg, author names and year), patients’ characteristics (eg, sample size, mean age, sex, and diseases), type of interventions and its components, setting of intervention, number of visits, PCNE MR category, method of communication with patients and physicians, and clinical outcomes.

Nonpharmacological interventions included education in lifestyle changes, medication use and disease; self‐monitoring of parameters; vitals assessment such as BP, capillary glycemia, or cholesterol measurements; and adherence interventions. Pharmacological interventions consisted of pharmacists suggesting modifications to treatment in detected drug‐related problems or only in CVD risk problems.^5^, ^6^, ^7^, ^8^, ^9^, ^10^

Two ambulatory settings were included. An ambulatory clinic (AC) is defined as a primary care center where health care is mostly provided by general practitioners but could also include specialized outpatient clinics.^15^ Community pharmacies (CPs) are legally approved establishments that supply prescription and nonprescription medicines and may provide professional pharmacy services and patient counselling while dispensing.^16^

### Quality Assessment

The revised Cochrane risk‐of‐bias tool for randomized controlled trials was used to identify the risk of bias. Studies were classified as having low risk, high risk, or some concerns of bias.^17^

### Statistical Analyses

Pairwise meta‐analyses of the studies were performed for the outcome measures whenever possible. These analyses were conducted using the software Comprehensive Meta‐Analysis version 3 (Biostat).

The random effect model was used with the inverse of the variance to obtain pooled effect sizes, and results were reported with a 95% CI and *P*<0.05. The calculation of 95% prediction intervals (PIs) was performed in preformatted sheets in Excel with the method described by Borenstein and Higgins using mean effect size and its variance (random effect weights), degrees of freedom, and Tau^2^ (estimation measure of the true effect size distribution) in log units (normal approximation).^18^, ^19^ PIs allow more informative inferences in meta‐analyses (eg, true treatment effects that can be expected in future settings), especially when there is large variation in the strength of the effect (high heterogeneity between studies).^14^, ^18^, ^19^

For the meta‐analyses of dichotomous data (therapeutic goals), the odds ratio (OR) was calculated. For the meta‐analyses of continuous outcomes, the differences between baseline and final values with the corresponding SDs reported by the individual studies (pre‐post correlation of 0.999) were used.^14^

For articles that reported 95% CI as a measure of uncertainty, SD was calculated using the size of the samples, the length of the CI, and the value from Student *t* distribution. When numeric data were insufficient to conduct the pooled analysis, a request was sent to the author by email. If the authors responded negatively or not at all, we excluded the article from the analyses.^18^, ^19^

The between‐trial heterogeneity was assessed using the inconsistency index value (I2 statistic) with ranges of <25% (low), 25–50% (moderate), 50–75% (high) and >75% (very high) heterogeneity. Sensitivity analyses were conducted together with analyses for publication and other bias (funnel and scatter plots, Failsafe N) to test the robustness of the results. Subgroup analyses considering setting and components of interventions were performed when possible.^14^

## Results

**Figure 1:**
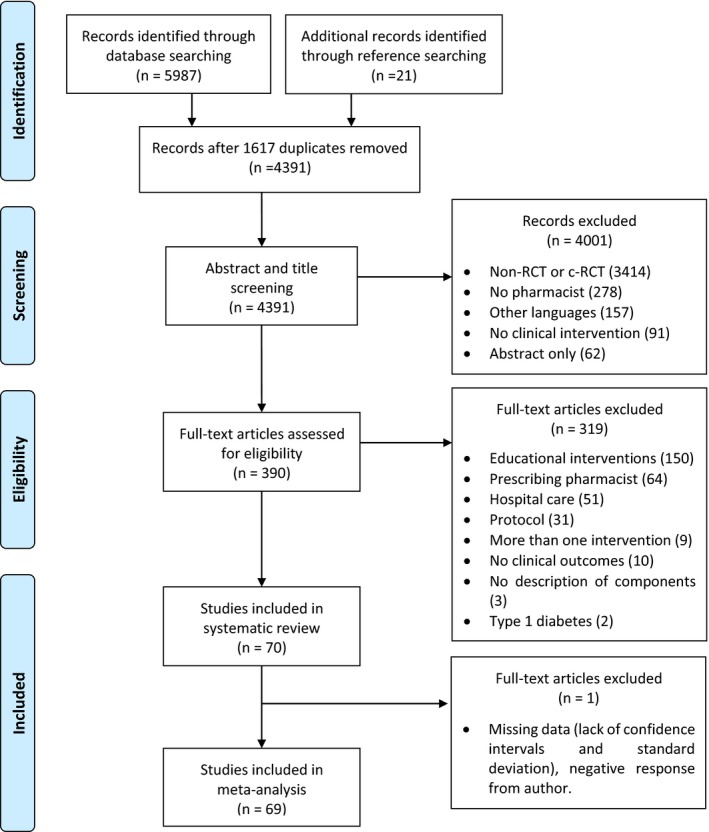
PRISMA flowchart for systematic review and meta‐analysis.^12^, ^13^. c‐RCT indicates cluster randomized controlled trial; RCT, randomized controlled trial.

Sixty‐nine studies reported data that could be included in the meta‐analyses (Figure 1). One study was excluded from these analyses because it lacked uncertainty data (and the author responded negatively). Forty‐five of these studies were undertaken in ACs and 24 in CPs. The total number of patients was 11 743, with 11 644 included in the meta‐analyses. Of these, 8014 patients were in ACs and 3630 in CPs, with a mean age of 60±7.2 years, and the percentage of men in the included studies was 43±8.8%, without differences between subgroups (Table 2). The mean follow‐up time was 8.35±4.44 months, and there were 5.21±2.52 contacts with patients in average. Most studies provided lifestyle and disease education, and 23 studies considered the opinion of each patient before changing pharmacotherapy. In 39 studies, pharmacists only implemented changes in medications for CVD risk (ignoring other medical conditions). In 48 studies, pharmacists assessed vitals during the interviews and provided self‐monitoring education in 38 studies.

**Table 2:** Included Studies Metadata

Lead Study Author and Date	Age (SD), y	No. of IG (% of Men)	No. of CG (% of Men)	MR	Goal Included	Continuous Outcomes								Visits	Mo	Contact			Components						
						SBP	DBP	HbA_1c_	FG	TC	LDL	HDL	Triglyceride			Physician	Patient	Specialist	Disease Education	Self‐Monitoring	Lifestyle Education	All DRP	Patient	Vitals	Risk of Bias
CPs																									
Amariles 2012^20^	63 (11)	356 (51)	358 (54)	2	BP, TC	X	X			X				6	8	W	I		X	X	X	X	X	X	L
Bajorek 2016^21^	71 (14)	10 (30)	11 (60)	2		X	X							4	12	W	I/P			X	X	X		X	H
Basheti 2016^22^	53 (16)	82 (47)	78 (47)	3		X	X		X				X	2	3	W	I		X		X	X		X	C
Chung 2014^23^	59 (9)	120 (42)	121 (46)	3	T2DM			X	X					4	12	I	I		X	X	X				C
Doucette 2009^24^	60 (12)	36 (48)	42 (46)	3				X			X			4	12	W	I		X	X	X			X	C
Fornos 2006^25^	64 (11)	56 (NI)	56 (NI)	2		X	X	X		X	X	X	X	12	12	W	I		X	X	X	X	X	X	C
Garcao 2002^26^	65 (10)	41 (34)	41 (22)	2	BP	X	X							6	6	W	I		X	X	X	X	X	X	C
Jahangard‐Rafsanjani 2014^27^	57 (8)	45 (51)	40 (48)	2		X	X	X						6	6	W	I	X	X	X	X	X	X	X	L
Kjeldsen 2014^28^	63 (10)	70 (61)	102 (62)	3		X								4	6	W	I		X	X	X	X	X	X	C
Krass 2007^29^	62 (11)	87 (51)	92 (51)	2	BP	X	X	X		X			X	5	6	W	I		X	X	X			X	C
Lugo De Ortellado 2008^30^	47 (8)	33 (31)	28 (31)	2		X	X							6	6	W	I		X	X	X	X	X	X	C
Nola 2000^31^	60 (10)	25 (46)	26 (36)	2	TC					X	X	X	X	5	6	W	I		X		X			X	L
Park 1996^32^	60 (10)	27 (50)	26 (48)	2	BP	X	X							6	6	W	I				X	X		X	C
Paulo 2016^33^	58 (3)	47 (43)	42 (48)	2		X	X	X	X	X	X	X	X	6	6	W	I		X	X	X	X	X	X	C
Paulos 2005^34^	64 (11)	23 (19)	19 (19)	2						X			X	5	4	W	I		X		X			X	C
Planas 2009^35^	65 (12)	25 (35)	15 (39)	2	BP	X								9	9	W	I				X	X		X	H
Robinson 2010^36^	65 (10)	78 (NI)	62 (NI)	2	BP	X	X							3	12	W	I		X		X			X	H
Skowron 2010^37^	60 (10)	28 (40)	56 (41)	2		X	X							12	6	W	I		X		X	X		X	C
Stewart 2014^38^	67 (12)	176 (48)	176 (55)	2		X	X							3	6	W	I		X	X	X			X	C
Taylor 2005^39^	65 (12)	53 (45)	46 (43)	2				X						7	9	W	I		X	X	X	X			H
Torres 2009^40^	68 (10)	183 (37)	183 (41)	2	BP	X	X							6	6	W	I		X	X	X	X	X	X	C
Villeneuve 2010^41^	61 (11)	108 (64)	117 (61)	2	TC	X	X		X	X	X	X	X	6	12	I	I				X				C
Wang 2011^42^	48 (9)	29 (52)	30 (47)	2		X	X							6	12	W	I		X	X	X	X	X	X	L
Zillich 2005^43^	65 (7)	64 (36)	61 (42)	2	BP	X	X							4	3	W/P	I/P		X	X	X		X	X	C
ACs																									
Abuloha 2016^44^	55 (10)	45 (42)	43 (42)	3				X	X					3	3	I	I	X	X	X	X				L
Aguiar 2016^45^	62 (8)	36 (31)	37 (35)	3	BP, T2DM	X	X	X			X			3	12	I	I/P	X	X	X	X	X	X		L
Al Mazroui 2009^46^	49 (8)	117 (71)	117 (68)	3	T2DM	X	X	X	X	X	X	X	X	4	12	I	I	X	X	X	X				C
Albsoul‐Younes 2011^47^	57 (10)	130 (47)	123 (48)	3	BP	X	X							9	9	I	I		X	X	X			X	L
Azevedo 2017^48^	63 (11)	33 (21)	30 (27)	3		X	X		X	X	X	X	X	8	6	W/I	I/P		X		X		X	X	H
Bogden 1997^49^	57 (12)	47 (34)	47 (19)	3	TC					X				12	6	I	I								C
Bogden 1998^50^	55 (8)	49 (41)	46 (43)	3	BP	X	X							6	6	I	I/P							X	H
Borenstein 2003^51^	62 (5)	98 (37)	99 (41)	3	BP	X								12	12	P	I	X			X			X	H
Carter 2008^52^	61 (12)	101 (42)	78 (46)	3	BP	X	X							6	9	I	I				X			X	C
Carter 2009^53^	59 (14)	192 (38)	210 (44)	3	BP	X	X							3	6	I	I							X	L
Chan 2012^54^	63 (10)	51 (59)	54 (52)	3	BP, T2DM, TC	X	X	X		X	X	X	X	4	9	W	I		X		X	X		X	L
Chen 2016^55^	72 (6)	50 (51)	50 (49)	3				X						2	6	I	I/P								L
Choe 2005^56^	52 (10)	36 (49)	29 (46)	3				X						6	12	I	I/P		X	X	X	X			L
Clifford 2002^57^	60 (12)	48 (48)	25 (58)	3				X						4	6	I	I	X	X		X				C
Clifford 2005^58^	70 (8)	92 (48)	88 (57)	2		X	X	X	X	X		X	X	3	12	W	I/P		X	X	X	X	X	X	L
de Castro 2015^59^	61 (10)	30 (31)	34 (38)	3		X	X							5	6	I	I	X	X	X	X	X	X	X	C
Ebid 2014^60^	54 (13)	140 (51)	140 (48)	3	BP	X	X							3	3	I	I	X	X	X	X	X		X	L
Firminho 2015^61^	60 (9)	26 (24)	30 (24)	3		X	X		X	X	X	X	X	6	9	W	I		X	X	X	X	X	X	L
Green 2008^62^	59 (9)	237 (56)	247 (55)	3	BP	X	X							1	12	W	W		X	X	X		X		C
Hammad 2011^63^	57 (10)	110 (36)	89 (39)	3		X	X		X			X	X	6	6	I	I		X	X	X				L
Hedegaard 2015^64^	61 (4)	231 (59)	285 (59)	3	BP	X	X							4	12	I	I/P	X	X		X				L
Hunt 2008^65^	68 (12)	142 (37)	130 (34)	3	BP	X	X							4	12	W	I		X		X			X	L
Jacobs 2012^66^	63 (11)	72 (68)	92 (55)	3	BP, T2DM, TC	X	X	X						3	12	W/I	W/I		X	X	X	X	X	X	L
Jameson 2010^67^	49 (11)	52 (49)	51 (49)	3	T2DM			X						6	12	W	I/P		X	X	X				L
Jarab 2012^68^	64 (10)	77 (57)	79 (56)	3	BP, T2DM, TC	X	X	X	X	X	X	X	X	3	6	I	I/P		X	X	X	X			L
Korcegez 2017^69^	62 (10)	75 (23)	77 (26)	3	BP, T2DM	X	X	X	X	X	X	X	X	5	12	W	I		X	X	X	X	X	X	C
Lee 2009^70^	62 (11)	58 (59)	60 (43)	3						X	X	X	X	3	6	W/I	I/P	X	X		X				L
Morgado 2011^71^	59 (12)	76 (45)	99 (35)	3	BP	X	X							3	9	I	I		X	X	X	X	X		L
Mourao 2013^72^	61 (10)	50 (32)	50 (34)	3		X	X	X	X	X	X	X	X	6	6	W	I		X	X	X	X		X	L
Obreli‐Neto 2011^73^	65 (6)	97 (37)	97 (38)	3	BP, T2DM, TC	X	X	X	X	X	X	X	X	6	36	W	I		X	X	X	X	X	X	C
Okamoto 2001^74^	62 (11)	164 (56)	166 (46)	3		X	X							2	6	W/I	I				X			X	L
Plaster 2012^75^	55 (12)	34 (29)	29 (49)	3		X	X		X	X	X	X	X	6	6	W	I		X	X	X	X	X	X	C
Polgreen 2015^76^	61 (1)	401 (40)	224 (40)	3	BP	X	X							6	9	I	I/P		X		X			X	L
Rothman 2005^77^	55 (12)	112 (44)	105 (44)	3		X	X	X		X				12	12	W	I/P		X		X			X	L
Sanchez‐Guerra 2018^78^	63 (7)	31 (32)	29 (31)	3		X	X							6	6	W	I		X			X			L
Scott 2006^79^	52 (16)	64 (42)	67 (36)	3	BP, T2DM, TC									7	9	I	I			X	X				L
Shao 2017^80^	59 (10)	100 (51)	99 (48)	3	BP, T2DM	X	X	X	X	X	X	X	X	3	6	W	I/P	X	X	X	X				C
Simpson 2011^81^	59 (12)	129 (44)	131 (42)	3	BP	X	X	X		X	X	X	X	2	6	I	I							X	L
Sookaneknun 2004^82^	63 (6)	118 (39)	117 (55)	3	BP	X	X							6	6	W	I				X		X	X	L
Tahaineh 2011^83^	53 (8)	73 (47)	52 (41)	3	TC									4	6	W	I				X			X	H
Taylor 2003^84^	66 (10)	24 (36)	29 (28)	3	BP, T2DM, TC									4	12	W/I	I		X		X	X			L
Tobari 2010^85^	62 (8)	66 (63)	66 (68)	3	BP	X	X							6	6	W/I	I		X	X	X				C
Villa 2009^86^	54 (8)	85 (36)	57 (53)	3						X	X	X	X	3	8	W/I	I		X		X		X	X	C
Wal 2013^87^	60 (9)	54 (47)	48 (52)	3		X	X							3	6	I	I/P	X	X		X	X		X	C
Wishah 2014^88^	53 (8)	52 (39)	54 (48)	3				X	X	X	X	X	X	3	6	W/I	I/P		X		X				C
Oparah 2009^a^, ^89^	55 (9)	50 (46)	49 (47)	3		X	X		X					12	3	W	I	X	X		X		X	X	H

ACs indicates ambulatory clinics; BP, blood pressure; C, some concerns; CG, control group; CPs, community pharmacies; DRPs, intervention in all drug‐related problems found; H, high risk; HbA_1c_, glycated hemoglobin; HDL, high‐density lipoprotein cholesterol; I, face‐to‐face interview; IG, intervention group; L, low risk; LDL, low‐density lipoprotein cholesterol; MR, Pharmaceutical Care Network Europe medication review category; NI, not informed; P, phone interview; T2DM, type 2 diabetes mellitus; TC, total cholesterol; W, written message (email or letter).

a Excluded from meta‐analysis for lacking baseline values.

### Risk of Bias

Sixty‐one of the 69 studies included in the meta‐analysis presented low risk or some concerns about bias. The main issues were the impossibility to blind patients to the intervention and the lack of details in the randomization process. Eight studies had a high risk of bias, mostly because of indefinite randomization and the lack of blinded process in the assessment of clinical outcomes. The effect of excluding high‐risk articles from the analyses was explored for each outcome.^20^, ^21^, ^22^, ^23^, ^24^, ^25^, ^26^, ^27^ Table S2 presents individual risk‐of‐bias analysis.

### Clinical Outcomes and Components

**Figure 2:**
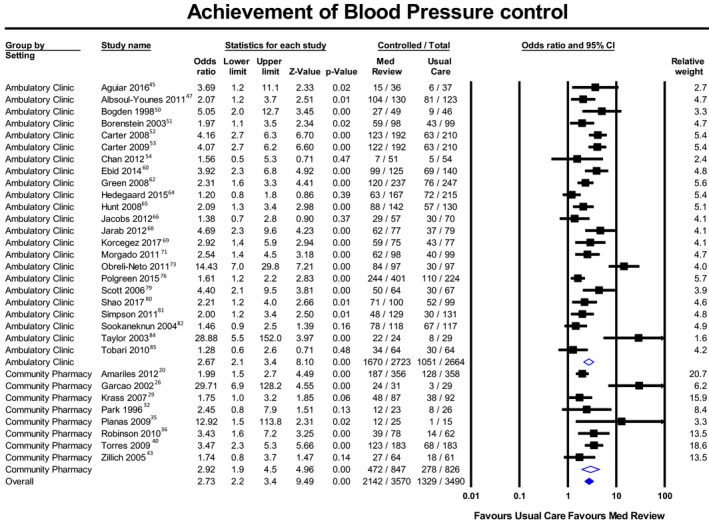
Meta‐analysis of patients reaching blood pressure control with medication reviews or usual care. Values in odds ratios with 95% CIs.

**Figure 3:**
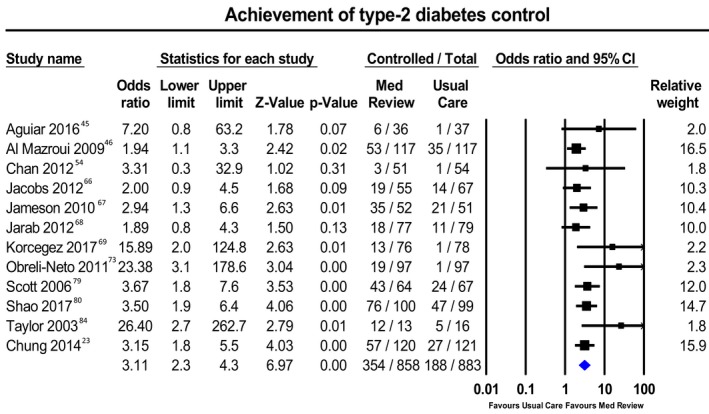
Meta‐analysis of patients with type 2 diabetes mellitus reaching glycated hemoglobin <7% with medication reviews or usual care. Values in odds ratios with 95% CIs.

Figures 2, 3 through 4 and Tables 3, 4 through 5 present clinical outcomes overall and by individual setting. Figures S1 through S8 contain additional forest plots for each clinical outcome and Table S3 shows the effect of each individual component and type of MR.

**Figure 4:**
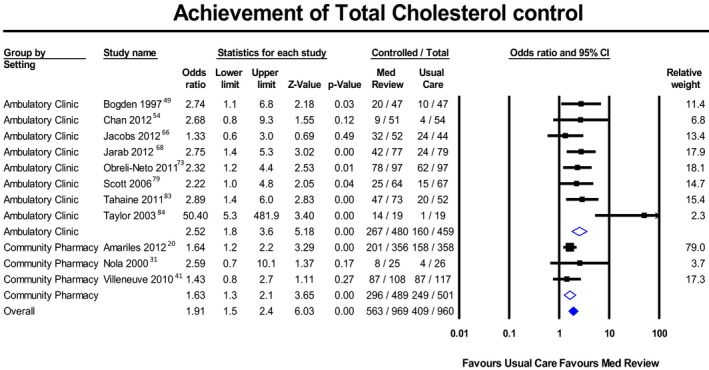
Meta‐analysis of patients reaching cholesterol control with medication reviews or usual care. Values in odds ratios with 95% CIs.

**Table 3:** Pooled Analysis of Hypertension Outcomes

Outcome	Analysis		Studies (No. of Patients)	Effect Size	95% CI	*I* ^2^, %	95% PI
BP control (OR)	Overall		31 (7031)	2.73	2.20–3.36^a^	71	1.05–7.08^a^
	Setting	AC	23 (5332)	2.67	2.11–3.39^a^	74	0.97–7.49
		CP	8 (1699)	2.92	1.91–4.46^a^	66	0.86–9.92
	Sample size	Excluding N <100^26^, ^32^, ^35^, ^45^, ^50^, ^84^	25 (6635)	2.43	2.02–2.93^a^	70	1.04–5.69^a^
	RoB	Excluding high^35^, ^36^, ^50^, ^51^, ^82^	26 (6324)	2.74	2.18–3.44^a^	73	1.02–7.39^a^
	Outliers	Excluding OR>20^26^, ^84^	29 (6896)	2.51	2.11–3.07^a^	67	1.08–5.82^a^
SBP, mm Hg	Overall		52 (9935)	−8.50	−9.66 to −7.34^a^	99	−19.0 to 1.68
	Setting	AC	33 (6816)	−8.34	−10.1 to −6.61^a^	99	−18.8 to 2.02
		CP	19 (3119)	−8.64	−10.2 to −7.07^a^	99	−16.0 to −1.26^a^
	Sample size	Excluding N <100^21^, ^26^, ^27^, ^30^, ^32^, ^33^, ^35^, ^37^	36 (8887)	−7.53	−9.17 to −5.89^a^	99	−17.8 to 2.76
	RoB	Excluding high^21^, ^35^, ^36^, ^39^, ^48^, ^50^, ^51^	45 (9144)	−7.94	−9.45 to −6.42^a^	99	−18.5 to 2.57
	Outliers	Excluding >20 mm Hg decrease in SBP^30^, ^35^, ^73^	49 (9640)	−7.54	−8.72 to −6.54^a^	99	−15.3 to −0.27^a^
DBP, mm Hg	Overall		49 (9526)	−3.68	−4.45 to −2.92^a^	99	−9.56 to 2.20
	Setting	AC	32 (6619)	−4.53	−5.75 to −3.32^a^	99	−11.8 to 2.74
		CP	17 (2907)	−3.13	−4.11 to −2.14^a^	99	−7.60, 1.34
	Sample size	Excluding N <100^21^, ^26^, ^27^, ^30^, ^32^, ^33^, ^37^, ^42^	34 (8518)	−3.85	−4.85 to −2.85^a^	99	−9.98 to 2.28
	RoB	Excluding high^21^, ^36^, ^48^, ^50^, ^82^	44 (8972)	−3.78	−4.65 to −2.91^a^	99	−9.74 to 2.18
	Outliers	Excluding >10 mm Hg decrease in DBP^50^, ^73^	47 (9237)	−3.72	−4.50 to −2.94^a^	99	−9.24 to 1.80

AC indicates ambulatory clinic; BP, blood pressure; CP, community pharmacy; DBP, diastolic blood pressure; N, total number of patients; OR, odds ratio; PI, prediction interval; RoB, risk of bias; SBP, systolic blood pressure.

a Statistical significance.

**Table 4:** Pooled Analysis of T2DM Outcomes

Outcome	Analysis		Studies (Patients)	Effect Size	95% CI	*I* ^2^, %	95% PI
T2DM control (OR)	Overall		12 (1805)	3.11	2.26–4.27^a^	30	1.48–6.52^a^
	Setting	Excluding CP	11 (1564)	3.18	2.18–4.65^a^	36	1.27–8.00^a^
	Sample size	Excluding N <100^45^, ^84^	10 (1679)	2.89	2.16–3.87^a^	22	1.58–5.27^a^
	Outliers	Excluding OR >15^69^, ^73^, ^84^	9 (1406)	2.71	2.11–3.47^a^	0	2.01–3.65^a^
HbA_1c_, %	Overall		25 (3452)	−0.81	−0.99 to −0.64^a^	99	−1.78 to 0.15
	Setting	AC	18 (2569)	−0.93	−1.17 to −0.69^a^	99	−2.05 to 0.19
		CP	7 (833)	−0.69	−0.94 to −0.45^a^	99	−1.57 to 0.19
	Sample size	Excluding N <100^24^, ^27^, ^33^, ^39^, ^44^, ^45^, ^56^, ^57^	17 (2802)	−0.99	−1.25 to −0.74^a^	99	−2.16 to 0.18
	Outliers	Excluding >1.5% decrease^46^	24 (3218)	−0.84	−0.97 to −0.70^a^	99	−1.51 to −0.13^a^
Fasting glucose, mg/dL	Overall		17 (2505)	−28.8	−38.1, −19.6^a^	99	−70.9, 13.2
	Setting	AC	13 (1790)	−30.9	−41.0 to −20.9^a^	99	−73.0 to 11.2
		CP	4 (715)	−18.2	−41.1 to 4.50	99	−13.0 to 94.0
	Sample size	Excluding N <100^33^, ^44^, ^48^, ^61^, ^75^	12 (2146)	−27.8	−37.1 to −18.5^a^	99	−65.8 to 10.2
	RoB	Excluding high^48^	16 (2442)	−28.3	−37.8 to −18.8^a^	99	−70.7 to 14.1
	Outliers	Excluding >50 mg/dL decrease^23^, ^68^, ^75^, ^88^	13 (1939)	−20.3	−27.9 to −12.7^a^	99	−51.8 to 11.2

AC indicates ambulatory clinic; CP, community pharmacy; HbA_1c_, glycated hemoglobin; OR, odds ratio; PI, prediction interval; RoB, risk of bias; T2DM, type 2 diabetes mellitus.

a Statistical significance.

**Table 5:** Pooled Analysis of Dyslipidemia Outcomes

Outcome	Analysis		Studies (Patients)	Effect Size	95% CI	*I* ^2^, %	95% PI
TC control (OR)	Overall		11 (2012)	1.91	1.55–2.35*	31	1.05–3.46*
	Setting	AC	8 (1022)	2.52	1.78–3.58*	26	1.18–5.40*
		CP	3 (990)	1.63	1.25–2.12*	0	0.29–8.97
	Sample size	Excluding N <100^31^, ^49^, ^84^	8 (1814)	1.87	1.68–2.90*	0	1.01–3.50*
	Outliers	Excluding OR >10^84^	10 (1959)	1.92	1.58–2.34*	0	1.53–2.42*
TC, mg/dL	Overall		24 (3851)	−14.3	−18.2 to −10.5*	99	−36.3 to 7.63
	Setting	AC	17 (2439)	−18.1	−23.2 to −12.9*	99	−41.6 to 5.52
		CP	7 (1412)	−9.73	−15.5 to −3.99*	99	−29.2 to 9.79
	Sample size	Excluding N <100^31^, ^33^, ^34^, ^48^, ^49^, ^61^, ^75^	17 (3393)	−14.7	−19.3 to −10.1*	99	−35.9 to 6.42
	RoB	Excluding high^48^	23 (3788)	−14.4	−18.3 to −10.5*	99	−36.1 to 7.28
	Outliers	Excluding >30 mg/dL decrease^46^, ^49^, ^68^	21 (3367)	−13.3	−16.7 to −10.0*	99	−29.7 to 3.06
LDL‐C, mg/dL	Overall		20 (2576)	−10.3	−12.1 to −8.57*	99	−23.9 to 3.31
	Setting	AC	15 (2021)	−15.3	−18.9 to −11.7*	99	−31.0 to 0.40
		CP	5 (555)	−8.80	−10.8 to −6.82*	96	−16.4 to −1.17*
	Sample size	Excluding N <100^24^, ^31^, ^33^, ^45^, ^48^, ^61^, ^75^	13 (2103)	−15.6	−18.7 to −12.4*	99	−28.6 to −2.52*
	RoB	Excluding high^48^	19 (2513)	−13.7	−16.6 to −10.7*	99	−27.7 to 0.38
	Outliers	Excluding >25 mg/dL decrease^46^, ^75^	18 (2279)	−12.1	−14.9 to −9.37*	99	−24.9 to 0.64
HDL‐C, mg/dL	Overall		20 (2804)	0.90	0.40–1.40*	99	−10.2 to 12.0
	Setting	AC	16 (2327)	4.07	1.66–6.49*	99	−6.80 to 15.0
		CP	4 (477)	0.76	0.26–1.27*	99	−1.49 to 3.02
	Sample size	Excluding N <100^31^, ^33^, ^48^, ^61^, ^75^	15 (2483)	2.87	0.58–5.17*	99	−7.21 to 13.0
	RoB	Excluding high^48^	19 (2741)	3.26	0.85–5.66*	99	−8.30 to 14.8
	Outliers	Excluding >10 mg/dL increase^73^, ^75^	18 (2376)	2.72	1.65–3.78*	99	−2.26 to 7.70
Triglycerides, mg/dL	Overall		23 (3185)	−29.7	−36.4 to −23.0*	99	−64.2 to 4.78
	Setting	AC	16 (2327)	−34.8	−43.8 to −25.8*	99	−74.4 to 4.83
		CP	7 (858)	−23.4	−33.4 to −13.4*	99	−57.7 to 11.0
	Sample size	Excluding N <100^31^, ^33^, ^34^, ^48^, ^61^, ^75^	17 (2821)	−30.2	−38.3 to −22.1*	99	−66.8 to 6.40
	RoB	Excluding high^48^	22 (3122)	−30.5	−37.5 to −23.5*	99	−65.1 to 4.08
	Outliers	Excluding >60 mg/dL decrease^34^, ^48^, ^68^, ^86^	19 (2782)	−24.3	−31.1 to −17.5*	99	−56.1 to 7.48

AC indicates ambulatory clinic; CP, community pharmacy; HDL‐C, high‐density lipoprotein cholesterol; LDL‐C, low‐density lipoprotein cholesterol; OR, odds ratio; PI, prediction interval; RoB, risk of bias; TC, total cholesterol.

Statistical significance.

### Hypertension

Table 3 presents pooled size effects for hypertension outcomes. Mean follow‐up time of the MR service was 8.49±4.99 months, with 5.28±2.59 patient visits. The meta‐analysis for overall BP control (31 studies; n=7031 patients) showed a statistically significant pooled OR of 2.73 (95% PI, 1.05–7.08) (Figure 2). Heterogeneity was high (*I* ^2^=71%) and the AC subgroup also had a significant PI.

Fifty‐two studies (n=9935 patients) were included in the analysis of systolic BP (SBP) (Figure S1). Heterogeneity was very high (*I* ^2^=99%) and resulted in significant PIs for the CP subgroup but not for the AC subgroup or overall.

For diastolic BP (DBP) (49 studies; n=9526 patients) heterogeneity was very high (*I* ^2^=99%), and PIs were not significant overall or in subgroups (Figure S2).

Excluding studies with a high risk of bias, small studies or outliers resulted in similar results for hypertension outcomes (Table 3).

### Type 2 Diabetes Mellitus

For diabetes mellitus studies, mean follow‐up time was 9.96±6.22 months, with 4.88±2.57 patient visits. The overall OR for achievement of T2DM control (12 studies; n=1805 patients) was 3.11 (95% PI, 1.48–6.52) (Figure 3). Only 1 CP article reported this outcome, and the AC subgroup showed a significant PI. Heterogeneity was moderate (*I* ^2^=30%). No article had a high risk of bias. Table 4 presents effect sizes for T2DM outcomes.

A total of 3452 patients with T2DM from 25 studies were included in the analysis of the differences in HbA_1c_ levels (Figure S3). There was very high heterogeneity (*I* ^2^=99%), which resulted in a nonsignificant PI. Subgroup analysis also showed no significant PI. No study had a high risk of bias.

In the fasting glucose analysis (17 studies; n=2505 patients) there was very high heterogeneity (*I* ^2^=99%) with nonsignificant PI (Figure S4).

Sensitivity analyses showed no differences except for the exclusion of 3 outliers in diabetes mellitus control, which reduced heterogeneity to 0%, and 1 outlier in HbA_1c_, which resulted in a significant PI overall (Table 4).

### Dyslipidemias

Table 5 presents dyslipidemia outcomes. Mean follow‐up time was 9.01±6.31 months, with 5.58±2.87 patient visits. Eleven studies (n=2012 patients) reported cholesterol goals (Figure 4), finding a significant OR of 1.91 (95% PI, 1.05–3.46), with moderate heterogeneity (*I* ^2^=31%). AC had a significant PI. There were no studies with a high risk of bias.

The analysis of total cholesterol had very high heterogeneity (*I* ^2^=99%) resulting in a nonsignificant PI (Figure S5). There was a significant difference between subgroups (Q=7.91, *P*=0.005), with ACs having a larger reduction in TC levels than CPs.

Very high heterogeneity (*I* ^2^=99%) was found in the low‐density lipoprotein cholesterol analysis, with a significant PI in the CP subgroup only (Figure S6). A statistical difference was observed between subgroups (Q=9.62, *P*=0.002) with a larger effect in ACs. CP analysis included 5 studies versus 15 in the AC subgroup.

For high‐density lipoprotein cholesterol (20 studies; n=2804 patients), there was very high heterogeneity (*I* ^2^=99%), which led to a nonsignificant PI (Figure S7). There was a significant difference between subgroups (Q=5.25, *P*=0.022), with a larger effect in ACs versus CPs, but none had statistical significance.

For triglyceride levels (23 studies; n=3185), a nonsignificant PI was observed with very high heterogeneity (*I* ^2^=99%) (Figure S8).

Excluding small studies or an outlier reduced heterogeneity to 0% and produced significant PI in the control of total cholesterol (Table 5).

## Discussion

To our knowledge, this is the first meta‐analysis for MRs that includes a high number of CVD outcomes and uses PIs to account for high heterogeneity. The inclusion of control of hypertension, T2DM, and dyslipidemia and continuous clinical outcomes allowed exploration of a multidimensional effect of the provision of MR by pharmacists. We included a high number of studies and accounted for multiple components of the intervention, exploring the effect of possible bias.

Settings presented significant differences in some outcomes, with the AC subgroup having larger effect sizes in cholesterol values and DBP. This subgroup had significant increases in the achievement of T2DM and TC goals with moderate heterogeneity. Continuous outcomes had high heterogeneity and nonsignificant PIs.

In contrast with community pharmacists, AC pharmacists could directly be part of clinical teams, which may help to increase the acceptance of interventions from physicians, thus increasing the impact of MR.^9^ This assumption could not be tested since only a small number of studies included acceptance rate. The AC group included more patients (almost twice), longer follow‐up times (3±7.3 months difference), and more studies in all outcomes than the CP subgroup. All of these elements could increase effects sizes and heterogeneity at the same time.^14^ More studies were undertaken in the AC setting, some with high effects (outliers); however, we found no differences in magnitude and significance of effects when removed from the analyses.

In CPs there were significant decreases in low‐density lipoprotein and SBP values. CP studies tended to be shorter and smaller than AC studies. All CP effects were more affected than ACs when accounting for a high risk of bias and publication bias, with fewer reporting the number of outcomes per study. The lower number of patients within each study could have lowered heterogeneity (increasing statistical significance) and effect sizes in almost all outcomes.^10^, ^14^

MR classification had significant differences between types 2 and 3, with advanced MRs providing larger effects in DBP, HbA_1c_, and lipids, but this significance is limited because of a small number of pairwise comparisons, and no study with type 1 MR classification resulted from the inclusion criteria (Table S3).^11^, ^14^ Most of the individual components of the MR service did not have significant effects on outcomes (Table S3). Assessment of BP during visits increased the effect in control of BP and SBP, as patients tend to improve compliance when they are tightly monitored.^1^, ^2^, ^3^

Decreasing cholesterol or BP values would be expected to happen faster and to require fewer visits than improving diabetes mellitus outcomes, but we found no differences in follow‐up times or the number of visits for the included studies or in regard to the observed effects.^1^, ^2^, ^3^ In hypertension, a significant increase overall in achievement of BP goals was found. Analyses show nonsignificant decreases in SBP and DBP (only CP achieved PI significance in SBP) but with high heterogeneity. Excluding small studies in the SBP analysis had no effect in the AC subgroup but decreased the effect in CPs and prevented significant PIs, which, together with an asymmetric funnel plot, suggested that there was a risk of publication bias in the CP subgroup (excluding outliers produced the same effect as they were mostly in CPs).^14^ The small effect in DBP could be explained by the fact that most included patients were older adults, who often have isolated systolic hypertension.^2^, ^3^

In T2DM, an overall significant increase in achieving HbA_1c_ goals was observed. Only 1 CP study reported this outcome despite most studies reporting HbA_1c_ percentages and being a key outcome, which prevented subgroup analysis.^9^ In dyslipidemia outcomes, the control of total cholesterol increased significantly overall and in ACs even while removing outliers or small studies, but not in continuous variables (except for low‐density lipoprotein cholesterol in CP with a low number of studies) as heterogeneity was high.

Previous reviews reported significant reductions in SBP, DBP, HbA_1c_, and cholesterol values, and our analysis reported a similar magnitude in clinical changes.^6^, ^7^, ^8^, ^9^, ^10^ However, we found that at a larger number of studies and when accounting for heterogeneity, statistical significance was lost in most continuous outcomes (as shown by nonsignificant PI). Nevertheless, our results support a significant effect in the control of these cardiovascular risk diseases by pharmacist‐led MRs, even when accounting for high heterogeneity.

There are only a limited number of studies that measure the impact of MR services by other health professionals. Nurses generally show lower effects than pharmacist‐led MRs in similar outcomes, but interventions that included both pharmacists and nurses seemed to provide better outcomes.^6^, ^7^, ^8^, ^9^, ^10^, ^90^, ^91^, ^92^, ^93^

Previous evidence has suggested that heterogeneity in pharmaceutical care studies could be accounted for by some major causes such as differences in sampling, patient demographic and clinical characteristics, differences in intervention components, and fidelity of the intervention.^9^, ^10^, ^96^, ^97^ We found that most studies had similar patient characteristics such as age, sex percentage, and baseline health conditions of patients. Interestingly, excluding outliers had no effect on the magnitude of point estimate or heterogeneity.

The effects of individual components of the MR service were examined, but the description of interventions was both vague and varied greatly. Most studies did not include key points such as acceptance rate for interventions and fidelity of the pharmacists to provide MR, which could have effects on outcomes.^96^, ^97^ The interaction between physicians and pharmacists was poorly described in many studies, therefore sensitivity analysis could not be performed. It would be optimal when generating evidence to have and use a standardized intervention that clearly defines the components and characteristics of the intervention, ie, dose and fidelity so that this source of heterogeneity could be ameliorated.^96^, ^97^

Intermediate and advanced MR services seem to provide benefits in controlling cardiovascular risk diseases as a result of many factors such as resolution of drug‐related problems, increase in medication adherence, simplification of therapies, and reduction of clinical inertia (common in cardiovascular conditions).^5^, ^6^, ^7^, ^8^, ^9^, ^10^, ^11^ We believe that the increase in control of hypertension, T2DM, and dyslipidemias of pharmacist‐led MR and its positive effects in most clinical outcomes support the implementation of this service, but more evidence is necessary regarding the in‐depth description of components to optimize its effects.

## Study Limitations

This study has several limitations. Moderate to high heterogeneity was observed, which represented a difficulty in establishing the true impact of MR. Individual effects of components of the MR interventions could not be adequately compared. Because of large variability in the number of reported components in many studies, combined effects meta‐regressions could not be performed, therefore paired analyses using means and *P* values for significance had to be used. These results could be biased by the combined accumulation of type I errors for the large number of studies, thus its results should be interpreted with caution.^14^ There could be some risk of bias as a result of the exclusion of languages other than English and Spanish, with differences in cultural and healthcare system organization.

## Conclusions

There is evidence to conclude that MRs provided by pharmacists may improve control of BP, cholesterol, and T2DM as significant effects sizes and PIs were found overall. We could not conclude that MR was better than usual care in most continuous clinical outcomes. Although effect sizes were positive with significant CIs for all analyses and settings, PI lacked significance in these outcomes. ACs had significant effects in the achievement of control of diabetes mellitus and high cholesterol, while CPs had significant decreases in SBP and low‐density lipoprotein cholesterol values, but larger studies are needed to further explore these differences. Advanced MRs in ACs could have larger effects in diabetes mellitus and cholesterol outcomes, but more evidence is needed. To ensure that there is optimization of research resources and for healthcare systems to adopt MR as usual practice, international standards should be set for the evaluation of MR services including defining in detail the target population and the MR intervention.

## Disclosures

None.

## Supporting information


**Table S1.** Complete Search Strategies
**Table S2.** Results of the Revised Cochrane Risk‐of‐Bias Tool for Randomized Controlled Trials and Cluster Randomized Trials
**Table S3.** Analysis of the Impact of Settings, Type of MR, and Components in All Outcomes
**Figure S1**. Raw mean difference on systolic blood pressure (SBP) in mm Hg.
**Figure S2**. Raw mean difference on diastolic blood pressure (DBP) in mm Hg.
**Figure S2**. Raw mean difference on diastolic blood pressure (DBP) in mm Hg.
**Figure S4**. Raw mean difference on fasting glucose in mg/dL.
**Figure S5.** Raw mean difference on total cholesterol in mg/dL.
**Figure S6.** Raw mean difference on low‐density lipoprotein (LDL) cholesterol in mg/dL.
**Figure S6.** Raw mean difference on low‐density lipoprotein (LDL) cholesterol in mg/dL .
**Figure S7**. Raw mean difference on high‐density lipoprotein (HDL) cholesterol in mg/dL.
**Figure S8**. Raw mean difference on triglycerides in mg/dL.
