# Supplementary material for: Systematic Review and Meta‐Analysis of Medication Reviews Conducted by Pharmacists on Cardiovascular Diseases Risk Factors in Ambulatory Care
Source: J Am Heart Assoc. 2019 Nov 12;8(22):e013627. doi: 10.1161/JAHA.119.013627 (PMC6915276; doi:10.1161/JAHA.119.013627)
Supplement: Supplementary file 1 — Table S1. Complete Search Strategies Table S2. Results of the Revised Cochrane Risk‐of‐Bias Tool for Randomized Controlled Trials and Cluster Randomized Trials Table S3. Analysis of the Impact of Settings, Type of MR, and Components in All Outcomes Figure S1. Raw mean difference on systolic blood pressure (SBP) in mm Hg. Figure S2. Raw mean difference on diastolic blood pressure (DBP) in mm Hg. Figure S2. Raw mean difference on diastolic blood pressure (DBP) in mm Hg. Figure S4. Raw mean difference on fasting glucose in mg/dL. Figure S5. Raw mean difference on total cholesterol in mg/dL. Figure S6. Raw mean difference on low‐density lipoprotein (LDL) cholesterol in mg/dL. Figure S6. Raw mean difference on low‐density lipoprotein (LDL) cholesterol in mg/dL . Figure S7. Raw mean difference on high‐density lipoprotein (HDL) cholesterol in mg/dL. Figure S8. Raw mean difference on triglycerides in mg/dL. [file JAH3-8-e013627-s001.pdf]

# Supplemental Material

**Table S1. Complete search strategies.**

| Database | Search details | Results |
|---|---|---|
| **MEDLINE (PubMed)** | ((((pharmaceutical services[MeSH Terms]) OR (pharmacists) OR "medication review" OR "pharmaceutical care") AND ((hypertension[MeSH Terms]) OR (diabetes mellitus, Type 2[MeSH Terms]) OR (cardiovascular) OR (cholesterol, LDL[MeSH Terms]) OR "blood pressure" OR diabetes OR hypertension))) AND ((primary OR ambulatory OR clinic OR outpatient OR pharmacies OR community)) | **3161** |
| **Web of Science (without MEDLINE)** | TI= ((pharmacists OR "pharmaceutical care" OR "medication review" OR "pharmaceutical services") AND (community OR ambulatory OR primary OR clinic) AND (cardiovascular OR hypertension OR diabetes OR cholesterol)) | **257** |
| **Embase (Ovid) (without MEDLINE)** | ((pharmacist or "medication review" or "pharmaceutical services" or "pharmaceutical care" or "pharmacy care" or pharmacists) and (community or clinic or primary) and (cardiovascular or hypertension or diabetes or cholesterol)).mp. | **331** |
| **Cochrane CENTRAL Library** | ((pharmacists OR "pharmaceutical care" OR "medication review" OR "pharmaceutical services") AND (community OR ambulatory OR primary OR clinic) AND (cardiovascular OR hypertension OR diabetes OR cholesterol)) | **1091** |
| **The Cumulative Index to Nursing and Allied Health Literature (CINAHL)** | ((pharmacists OR "pharmaceutical care" OR "medication review" OR "pharmaceutical services")) AND ((community OR ambulatory OR primary OR clinic)) AND ((cardiovascular OR hypertension OR diabetes OR cholesterol)) | **327** |

**Table S2. Results of the revised Cochrane risk of bias tool for randomized controlled trials and cluster randomized trials.**

| Study Name | Random | Individual allocation (clusters) | Deviations from the intervention | Missing data | Measure of outcome | Selection report | Others | Bias |
|---|---|---|---|---|---|---|---|---|
| Abuloha et al 2016[1] | L | | L | L | L | L | L | LOW |
| Aguiar et al 2016[2] | L | | L | L | L | L | C | LOW |
| Al Mazroui et al 2009[3] | C | | L | L | C | L | L | CONCERNS |
| Albsoul-Younes et al 2011[4] | L | | L | L | L | L | L | LOW |
| Amariles et al 2012[5] | L | L | L | L | L | L | L | LOW |
| Azevedo et al 2017[6] | C | | L | L | H | L | L | HIGH |
| Bajorek et al 2016[7] | C | C | H | L | C | L | L | HIGH |
| Basheti et al 2016[8] | L | | L | L | C | L | L | CONCERNS |
| Bogden et al 1997[9] | C | L | L | L | C | L | L | CONCERNS |
| Bogden et al 1998[10] | H | L | L | L | L | L | L | HIGH |
| Borenstein et al 2003[11] | C | | L | L | H | L | L | HIGH |
| Carter et al 2008[12] | L | C | L | L | C | L | L | CONCERNS |
| Carter et al 2009[13] | L | L | L | L | L | L | L | LOW |
| Chan et al 2012[14] | L | | L | L | L | L | L | LOW |
| Chen et al 2016[15] | L | | L | L | L | L | L | LOW |
| Choe et al 2005[16] | C | | L | L | L | C | L | CONCERNS |
| Chung et al 2014[17] | C | | L | L | C | L | L | CONCERNS |
| Clifford et al 2002[18] | L | | L | L | C | L | L | LOW |
| Clifford et al 2005[19] | C | | L | L | C | L | L | CONCERNS |
| de Castro et al 2015[20] | L | | L | L | C | L | L | LOW |
| Doucette et al 2009[21] | C | | L | L | C | L | L | CONCERNS |
| Ebid et al 2014[22] | C | | L | L | L | L | L | LOW |
| Firminho et al 2015[23] | L | | L | L | C | L | L | CONCERNS |
| Fornos et al 2006[24] | L | | L | L | C | L | L | CONCERNS |
| Garcao et al 2002[25] | C | | L | L | C | L | L | CONCERNS |
| Green et al 2008[26] | L | | L | L | L | L | L | LOW |
| Hammad et al 2011[27] | L | | L | L | L | L | L | LOW |

| | | | | | | | | | |
|---|---|---|---|---|---|---|---|---|---|
| Hedegaard et al 2015[28] | L | | L | L | L | L | L | LOW |
| Hunt et al 2008[29] | L | | L | L | L | L | C | LOW |
| Jacobs et al 2012[30] | L | | L | L | L | L | L | LOW |
| Jahangard-Rafsanjani et al 2014[31] | L | | L | L | L | L | L | LOW |
| Jameson et al 2010[32] | L | | L | L | L | L | L | LOW |
| Jarab et al 2012[33] | L | | L | L | C | C | L | CONCERNS |
| Kjeldsen et al 2014[34] | C | | L | L | C | L | L | CONCERNS |
| Korcegez et al 2017[35] | L | | L | L | L | L | L | LOW |
| Krass et al 2007[36] | C | C | L | L | C | L | L | CONCERNS |
| Lee et al 2009[37] | L | | L | L | L | L | L | LOW |
| Lugo De Ortellado et al 2008[38] | C | | L | L | C | L | L | CONCERNS |
| Morgado et al 2011[39] | L | | L | L | L | L | L | LOW |
| Mourao et al 2013[40] | L | | L | L | C | L | L | CONCERNS |
| Nola et al 2000[41] | L | | L | L | L | L | L | LOW |
| Obreli-Neto et al 2011[42] | L | | L | L | L | L | L | LOW |
| Okamoto et al 2001[43] | L | | L | L | C | L | C | CONCERNS |
| Oparah 2009[44] | L | | L | H | U | H | L | HIGH |
| Park et al 1996[45] | C | | L | L | C | L | L | CONCERNS |
| Paulo et al 2016[46] | C | | L | L | C | L | L | CONCERNS |
| Paulos et al 2005[47] | C | | L | L | C | L | L | CONCERNS |
| Planas et al 2009[48] | L | | L | L | C | H | L | HIGH |
| Plaster et al 2012[49] | L | | L | L | L | L | L | LOW |
| Polgreen et al 2015[50] | L | | L | L | L | L | L | LOW |
| Robinson et al 2010[51] | C | C | L | L | L | L | L | HIGH |
| Rothman et al 2005[52] | L | | L | L | L | L | L | LOW |
| Sanchez-Guerra et al 2018[53] | L | | L | L | L | L | L | LOW |
| Scott et al 2006[54] | L | | L | L | C | L | L | CONCERNS |
| Shao et al 2017[55] | L | | L | L | L | L | L | LOW |
| Simpson et al 2011[56] | L | | L | L | L | L | L | LOW |
| Skowron et al 2010[57] | C | C | L | L | C | L | L | CONCERNS |

| | | | | | | | | |
|---|---|---|---|---|---|---|---|---|
| Sookaneknun et al 2004[58] | L | | L | L | H | L | L | HIGH |
| Stewart et al 2014[59] | L | C | L | L | C | L | L | CONCERNS |
| Tahaine et al 2011[60] | L | | L | L | L | L | L | LOW |
| Taylor et al 2003[61] | C | | L | L | C | L | L | CONCERNS |
| Taylor et al 2016[62] | C | C | L | L | L | L | L | HIGH |
| Tobari et al 2010[63] | L | | L | L | C | L | L | CONCERNS |
| Torres et al 2009[64] | L | L | L | L | C | L | L | CONCERNS |
| Villa et al 2009[65] | C | | L | L | L | L | L | CONCERNS |
| Villeneuve et al 2010[66] | L | L | L | L | C | L | L | CONCERNS |
| Wal et al 2013[67] | L | | L | L | C | C | L | CONCERNS |
| Wang et al 2011[68] | L | | L | L | L | L | L | LOW |
| Wishah et al 2014[69] | L | | L | L | L | L | L | LOW |
| Zillich et al 2005[70] | L | C | L | L | C | L | L | CONCERNS |

C: some concerns; H: high risk; L: low risk.

**Table S3. Analysis of the impact of settings, type of MR and components in all outcomes.**

| Components | | BP Goal | T2D Goal | TC Goal | SBP | DBP | HbA1c | FG | TC | LDL-C | HDL-C | TG |
|---|---|---|---|---|---|---|---|---|---|---|---|---|
| **Type of MR** | 2 | 2.92 | N/A | 2.12 | -8.52 | -2.68 | -0.62 | -10.7 | -8.1 | -9.15 | 1.19 | -23.4 |
| | 3 | 2.68 | N/A | 2.52 | -8.41 | -4.65 | -0.94 | -31.6 | -19 | -14.8 | 4.21 | -34.8 |
| p value | | 0.73 | N/A | 0.58 | 0.92 | 0.01* | 0.03* | 0.04* | 0.01* | 0.01* | 0.02* | 0.11 |
| **Specialist Phys** | Y | 2.23 | 2.71 | N/A | -6.38 | -4.24 | -0.82 | -35.9 | -25 | -15.7 | 3.09 | -22.4 |
| | N | 2.82 | 3.67 | N/A | -8.97 | -4.61 | -0.97 | -29.4 | -17 | -15.2 | 4.3 | -34.3 |
| p value | | 0.40 | 0.44 | N/A | 0.15 | 0.81 | 0.62 | 0.42 | 0.12 | 0.94 | 0.48 | 0.62 |
| **Follow-up time** | ≤ 6 mo | 2.84 | 3.5 | 2.82 | -8.35 | -3.97 | -0.79 | -32.5 | -15 | -15.3 | 4.24 | -29.5 |
| | > 6 mo | 2.62 | 3.05 | 1.84 | -8.62 | -4.16 | -0.18 | -21.5 | -15 | -12 | 2.64 | -36.1 |
| p value | | 0.70 | 0.78 | 0.12 | 0.84 | 0.83 | 0.50 | 0.30 | 0.96 | 0.32 | 0.50 | 0.50 |
| **Number of visits** | ≤ 1v/mo | 2.32 | 3.15 | 3.32 | -8.42 | -3.93 | -0.94 | -32.2 | -15 | -16.7 | 6.64 | -34.7 |
| | > 1v/mo | 2.96 | 3.18 | 2.11 | -8.56 | -4.31 | -0.84 | -25 | -15 | -12.7 | 2.49 | -28.6 |
| p value | | 0.23 | 0.98 | 0.04* | 0.92 | 0.68 | 0.56 | 0.55 | 0.94 | 0.42 | 0.06 | 0.45 |
| **Disease Ed** | Y | 2.63 | 3.09 | 2.24 | -8.03 | -3.89 | -0.86 | -29.6 | -15 | -14.5 | 3.85 | -34.4 |
| | N | 2.97 | 3.67 | 2.57 | -10.3 | -4.75 | -0.84 | -2 | -15 | -6.89 | 0.6 | -22.4 |
| p value | | 0.57 | 0.67† | 0.62 | 0.25 | 0.27 | 0.98† | 0.01† | 0.95 | 0.01† | 0.01† | 0.01† |
| **Self** | Y | 2.88 | 3.09 | 1.84 | -8.22 | -4.12 | -0.86 | -29.3 | -15 | -14 | 4.25 | -34.2 |
| | N | 2.54 | 3.67 | 2.85 | -8.93 | -3.96 | -0.89 | -23.5 | -15 | -13.2 | 2.41 | -8 |
| p value | | 0.63 | 0.67† | 0.09 | 0.63 | 0.85 | 0.86 | 0.58 | 0.93 | 0.81 | 0.29 | 0.72 |
| **Lifestyle Ed** | Y | 2.66 | N/A | 2.33 | -8.43 | -3.97 | -0.86 | N/A | -15 | -14.2 | 3.7 | -32.4 |
| | N | 3.3 | N/A | 2.74 | -9.43 | -5.01 | -0.85 | N/A | -18 | -5 | 0.2 | -30 |
| p value | | 0.48† | N/A | 0.74† | 0.65 | 0.43 | 0.98† | N/A | 0.80† | 0.01† | 0.01† | 0.01† |
| **All DRP** | Y | 3.93 | 4.98 | 2.25 | -9.58 | -4.16 | -0.82 | -27.5 | -16 | -13.3 | 3.76 | -32.6 |
| | N | 2.22 | 2.87 | 2.63 | -7.19 | -3.91 | -0.9 | -28.3 | -15 | -14.1 | 3.28 | -16 |
| p value | | 0.01* | 0.23 | 0.58 | 0.10 | 0.78 | 0.69 | 0.91 | 0.86 | 0.78 | 0.81 | 0.87 |
| **Pat goals** | Y | 2.93 | 5.85 | 1.7 | -9.49 | -4.09 | -0.7 | -22.5 | -13 | -12.1 | 4.3 | -31.6 |
| | N | 2.62 | 2.91 | 2.73 | -7.77 | -4.03 | -0.94 | -31.9 | -17 | -14.9 | 2.88 | -30.2 |
| p value | | 0.62 | 0.31 | 0.02* | 0.24 | 0.94 | 0.12 | 0.31 | 0.23 | 0.40 | 0.44 | 0.18 |
| **Vitals assess** | Y | 2.76 | 5.85 | 2.3 | -9.49 | -4.15 | -0.75 | -23.5 | -13 | -13.1 | 3.64 | -39.5 |
| | N | 2.65 | 2.91 | 2.62 | -4.92 | -3.74 | -0.98 | -33 | -22 | -14.9 | 3.3 | -26.8 |
| p value | | 0.97 | 0.31 | 0.71 | 0.01* | 0.72 | 0.21 | 0.31 | 0.01* | 0.64 | 0.85 | 0.65 |

*: statistical significance; **†:** comparison made with 1 or 2 studies; All DRP: intervention in all drug related problems found; BP: blood pressure; Ed: education; HbA1c: glycated hemoglobin; HDL: high-density lipoprotein cholesterol; LDL: Low-density lipoprotein cholesterol; MR: Pharmaceutical Care Network Europe Medication review category; N/A: no studies to compare; Pat: patient; Phys: physician; Self: self-monitoring; T2D: type 2 diabetes; TC: total cholesterol; TG: triglycerides; v/mo: visits per month.

**Figure S1**. **Raw mean difference on Systolic Blood Pressure in millimetres of mercury.**

# Mean difference in Systolic Blood Pressure

| Group by Setting | Study name | Difference in means | Standard error | Lower limit | Upper limit | Z-Value | p-Value | Med Review | Usual Care | Relative weight |
|---|---|---|---|---|---|---|---|---|---|---|
| Ambulatory Clinic | Aguiar 2016 [2] | -8.10 | 0.2 | -8.5 | -7.7 | -41.4 | 0.00 | 36 | 37 | 3.1 |
| Ambulatory Clinic | Al Mazroui 2009 [3] | -3.20 | 0.8 | -4.7 | -1.7 | -4.2 | 0.00 | 117 | 117 | 3.0 |
| Ambulatory Clinic | Albsoul-Younes 2011 [4] | -5.50 | 0.1 | -5.7 | -5.3 | -58.2 | 0.00 | 130 | 123 | 3.1 |
| Ambulatory Clinic | Azevedo 2017 [6] | -17.20 | 0.9 | -18.9 | -15.5 | -19.8 | 0.00 | 33 | 30 | 3.0 |
| Ambulatory Clinic | Bogden 1998 [9] | -12.00 | 0.8 | -13.6 | -10.4 | -15.0 | 0.00 | 49 | 46 | 3.0 |
| Ambulatory Clinic | Borenstein 2003 [11] | -11.00 | 0.1 | -11.3 | -10.7 | -84.4 | 0.00 | 98 | 99 | 3.1 |
| Ambulatory Clinic | Carter 2008 [12] | -11.60 | 0.5 | -12.6 | -10.6 | -22.2 | 0.00 | 101 | 78 | 3.0 |
| Ambulatory Clinic | Carter 2009 [13] | -13.90 | 0.5 | -14.9 | -12.9 | -27.6 | 0.00 | 192 | 210 | 3.0 |
| Ambulatory Clinic | Chan 2012 [14] | -3.30 | 0.2 | -3.7 | -2.9 | -16.5 | 0.00 | 51 | 54 | 3.1 |
| Ambulatory Clinic | Clifford 2005 [19] | -7.00 | 1.1 | -9.1 | -4.9 | -6.6 | 0.00 | 92 | 88 | 2.9 |
| Ambulatory Clinic | de Castro 2015 [20] | -5.00 | 1.2 | -7.4 | -2.6 | -4.1 | 0.00 | 30 | 34 | 2.9 |
| Ambulatory Clinic | Ebid 2014 [22] | -5.90 | 0.1 | -6.2 | -5.6 | -41.3 | 0.00 | 140 | 140 | 3.1 |
| Ambulatory Clinic | Firminho 2015 [23] | -10.50 | 1.0 | -12.4 | -8.6 | -10.8 | 0.00 | 26 | 30 | 3.0 |
| Ambulatory Clinic | Green 2008 [26] | -9.30 | 0.1 | -9.4 | -9.2 | -165.7 | 0.00 | 237 | 247 | 3.1 |
| Ambulatory Clinic | Hammad 2011 [27] | -4.60 | 0.4 | -5.5 | -3.7 | -10.5 | 0.00 | 110 | 89 | 3.0 |
| Ambulatory Clinic | Hedegaard 2015 [28] | -1.50 | 0.2 | -1.8 | -1.2 | -8.6 | 0.00 | 231 | 285 | 3.1 |
| Ambulatory Clinic | Hunt 2008 [29] | -5.00 | 0.7 | -6.4 | -3.6 | -7.2 | 0.00 | 142 | 130 | 3.0 |
| Ambulatory Clinic | Jacobs 2012 [30] | -10.60 | 0.4 | -11.3 | -9.9 | -28.1 | 0.00 | 72 | 92 | 3.0 |
| Ambulatory Clinic | Jarab 2012 [33] | -6.90 | 0.1 | -7.1 | -6.7 | -57.7 | 0.00 | 77 | 79 | 3.1 |
| Ambulatory Clinic | Korcegez 2017 [35] | -8.20 | 0.6 | -9.5 | -6.9 | -12.6 | 0.00 | 75 | 77 | 3.0 |
| Ambulatory Clinic | Morgado 2011 [39] | -6.80 | 0.2 | -7.2 | -6.4 | -37.4 | 0.00 | 76 | 99 | 3.1 |
| Ambulatory Clinic | Mourao 2013 [40] | -11.50 | 0.2 | -11.9 | -11.1 | -53.7 | 0.00 | 50 | 50 | 3.1 |
| Ambulatory Clinic | Obreli-Neto 2011 [42] | -22.60 | 0.1 | -22.9 | -22.3 | -164.9 | 0.00 | 97 | 97 | 3.1 |
| Ambulatory Clinic | Okamoto 2001 [43] | -7.90 | 0.3 | -8.4 | -7.4 | -30.9 | 0.00 | 164 | 166 | 3.1 |
| Ambulatory Clinic | Plaster 2012 [49] | -10.00 | 0.3 | -10.5 | -9.5 | -39.1 | 0.00 | 34 | 29 | 3.1 |
| Ambulatory Clinic | Polgreen 2015 [50] | -5.80 | 0.2 | -6.3 | -5.3 | -24.4 | 0.00 | 401 | 224 | 3.1 |
| Ambulatory Clinic | Rothman 2005 [52] | -15.00 | 0.1 | -15.3 | -14.7 | -110.4 | 0.00 | 112 | 105 | 3.1 |
| Ambulatory Clinic | Sanchez-guerra 2018 [53] | -7.00 | 0.3 | -7.7 | -6.3 | -20.6 | 0.00 | 31 | 29 | 3.0 |
| Ambulatory Clinic | Shao 2017 [55] | -3.90 | 0.6 | -5.1 | -2.7 | -6.5 | 0.00 | 100 | 99 | 3.0 |
| Ambulatory Clinic | Simpson 2011 [56] | -4.90 | 0.4 | -5.7 | -4.1 | -11.6 | 0.00 | 129 | 131 | 3.0 |
| Ambulatory Clinic | Sookaneknun 2004 [58] | -5.70 | 0.5 | -6.7 | -4.7 | -11.7 | 0.00 | 118 | 117 | 3.0 |
| Ambulatory Clinic | Tobari 2010 [63] | -1.50 | 0.1 | -1.7 | -1.3 | -15.1 | 0.00 | 66 | 66 | 3.1 |
| Ambulatory Clinic | Wal 2013 [67] | -12.20 | 0.4 | -13.0 | -11.4 | -31.8 | 0.00 | 54 | 48 | 3.0 |
| Ambulatory Clinic | | -8.34 | 0.9 | -10.1 | -6.6 | -9.5 | 0.00 | 3471 | 3345 | |
| Community Pharmacy | Amariles 2012 [5] | -6.50 | 0.2 | -6.9 | -6.1 | -31.9 | 0.00 | 356 | 358 | 5.5 |
| Community Pharmacy | Bajorek 2016 [7] | -13.00 | 1.2 | -15.4 | -10.6 | -10.5 | 0.00 | 27 | 11 | 4.9 |
| Community Pharmacy | Basheti 2016 [8] | -12.40 | 0.6 | -13.5 | -11.3 | -22.2 | 0.00 | 82 | 78 | 5.4 |
| Community Pharmacy | Fornos 2006 [24] | -9.00 | 0.6 | -10.1 | -7.9 | -16.0 | 0.00 | 56 | 56 | 5.4 |
| Community Pharmacy | Garcao 2002 [25] | -18.40 | 1.5 | -21.2 | -15.6 | -12.7 | 0.00 | 41 | 41 | 4.7 |
| Community Pharmacy | Jahangard-Rafsanjani 2014 [31] | -3.00 | 0.2 | -3.5 | -2.5 | -12.9 | 0.00 | 45 | 40 | 5.5 |
| Community Pharmacy | Kjeldsen 2014 [34] | -5.30 | 0.3 | -5.8 | -4.8 | -19.3 | 0.00 | 70 | 102 | 5.5 |
| Community Pharmacy | Krass 2007 [36] | -4.80 | 0.4 | -5.5 | -4.1 | -13.7 | 0.00 | 87 | 92 | 5.5 |
| Community Pharmacy | Lugo De Ortellado 2008 [38] | -25.30 | 1.1 | -27.4 | -23.2 | -23.5 | 0.00 | 33 | 28 | 5.0 |
| Community Pharmacy | Park 1996 [45] | -13.00 | 1.9 | -16.7 | -9.3 | -6.9 | 0.00 | 23 | 26 | 4.2 |
| Community Pharmacy | Paulo 2016 [46] | -1.60 | 0.2 | -2.0 | -1.2 | -8.7 | 0.00 | 47 | 42 | 5.5 |
| Community Pharmacy | Planas 2009 [48] | -20.10 | 1.3 | -22.7 | -17.5 | -15.2 | 0.00 | 25 | 15 | 4.8 |
| Community Pharmacy | Robinson 2010 [51] | -7.10 | 0.1 | -7.3 | -6.9 | -64.0 | 0.00 | 78 | 62 | 5.5 |
| Community Pharmacy | Skowron 2010 [57] | -1.00 | 0.3 | -1.7 | -0.3 | -2.9 | 0.00 | 28 | 56 | 5.5 |
| Community Pharmacy | Stewart 2014 [59] | -8.70 | 0.5 | -9.6 | -7.8 | -18.1 | 0.00 | 176 | 176 | 5.4 |
| Community Pharmacy | Torres 2009 [64] | -6.20 | 0.2 | -6.6 | -5.8 | -28.2 | 0.00 | 183 | 183 | 5.5 |
| Community Pharmacy | Villeneuve 2010 [66] | -1.30 | 0.1 | -1.5 | -1.1 | -16.1 | 0.00 | 108 | 117 | 5.5 |
| Community Pharmacy | Wang 2011 [68] | -9.00 | 0.8 | -10.6 | -7.4 | -11.2 | 0.00 | 29 | 30 | 5.2 |
| Community Pharmacy | Zillich 2005 [70] | -4.40 | 0.2 | -4.8 | -4.0 | -20.5 | 0.00 | 64 | 61 | 5.5 |
| Community Pharmacy | | -8.64 | 0.8 | -10.2 | -7.1 | -10.8 | 0.00 | 1558 | 1574 | |
| Overall | | -8.50 | 0.6 | -9.7 | -7.3 | -14.4 | 0.00 | 5029 | 4919 | |

Difference in means and 95% CI

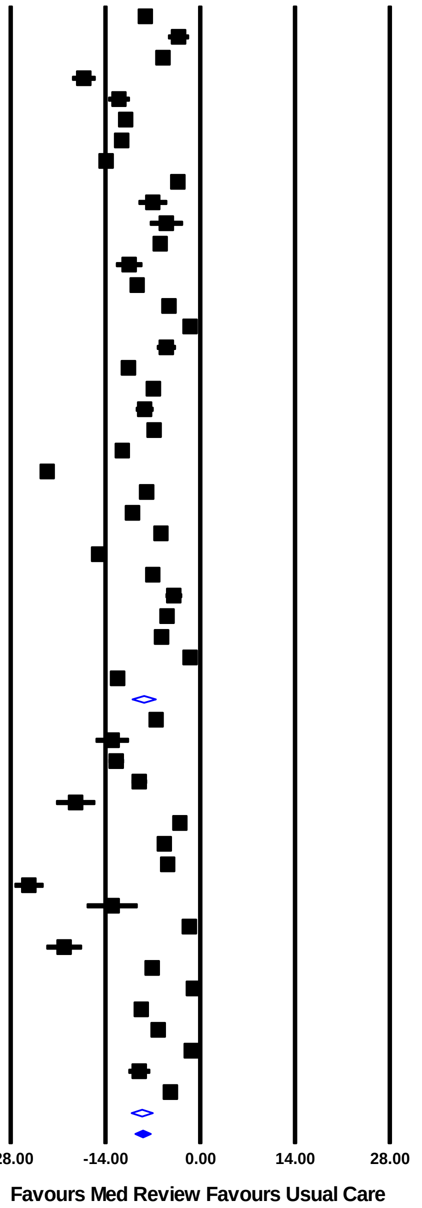


CI: confidence interval.

**Figure S2**. **Raw mean difference on Diastolic Blood Pressure in millimetres of mercury.**

# Mean difference in Diastolic Blood Pressure

| Group by Setting | Study name | Difference in means | Standard error | Lower limit | Upper limit | Z-Value | p-Value | Med Review | Usual Care | Relative weight |
|---|---|---|---|---|---|---|---|---|---|---|
| | | **Statistics for each study** | | | | | | **Sample size** | | |
| Ambulatory Clinic | Aguiar 2016[2] | -3.10 | 0.12 | -3.34 | -2.86 | -25.24 | 0.00 | 36 | 37 | 3.1 |
| Ambulatory Clinic | Al Mazroui 2009[3] | -9.10 | 0.17 | -9.43 | -8.77 | -54.81 | 0.00 | 117 | 117 | 3.1 |
| Ambulatory Clinic | Albsoul-Younes 2011[4] | -3.30 | 0.33 | -3.94 | -2.66 | -10.08 | 0.00 | 130 | 123 | 3.1 |
| Ambulatory Clinic | Azevedo 2017[6] | -9.70 | 0.57 | -10.81 | -8.59 | -17.11 | 0.00 | 33 | 30 | 3.1 |
| Ambulatory Clinic | Bogden 1998[9] | -11.00 | 0.47 | -11.93 | -10.07 | -23.22 | 0.00 | 49 | 46 | 3.1 |
| Ambulatory Clinic | Carter 2008[12] | -3.30 | 0.28 | -3.85 | -2.75 | -11.71 | 0.00 | 101 | 78 | 3.1 |
| Ambulatory Clinic | Carter 2009[13] | -5.20 | 0.16 | -5.52 | -4.88 | -32.08 | 0.00 | 192 | 210 | 3.1 |
| Ambulatory Clinic | Chan 2012[14] | -2.10 | 0.10 | -2.29 | -1.91 | -21.68 | 0.00 | 51 | 54 | 3.1 |
| Ambulatory Clinic | Clifford 2005[19] | -3.00 | 0.15 | -3.30 | -2.70 | -19.76 | 0.00 | 92 | 88 | 3.1 |
| Ambulatory Clinic | de Castro 2015[20] | -2.00 | 0.28 | -2.54 | -1.46 | -7.23 | 0.00 | 30 | 34 | 3.1 |
| Ambulatory Clinic | Ebid 2014[22] | -6.80 | 0.09 | -6.97 | -6.63 | -76.72 | 0.00 | 140 | 140 | 3.1 |
| Ambulatory Clinic | Firminho 2015[23] | -6.70 | 0.42 | -7.51 | -5.89 | -16.13 | 0.00 | 26 | 30 | 3.1 |
| Ambulatory Clinic | Green 2008[26] | -3.60 | 0.68 | -4.93 | -2.27 | -5.31 | 0.00 | 237 | 247 | 3.0 |
| Ambulatory Clinic | Hammad 2011[27] | -7.00 | 0.06 | -7.12 | -6.88 | -114.94 | 0.00 | 110 | 89 | 3.1 |
| Ambulatory Clinic | Hedegaard 2015[28] | -0.20 | 0.09 | -0.39 | -0.01 | -2.12 | 0.03 | 231 | 285 | 3.1 |
| Ambulatory Clinic | Hunt 2008[29] | -1.00 | 0.39 | -1.77 | -0.23 | -2.53 | 0.01 | 142 | 130 | 3.1 |
| Ambulatory Clinic | Jacobs 2012[30] | -6.70 | 0.28 | -7.26 | -6.14 | -23.52 | 0.00 | 72 | 92 | 3.1 |
| Ambulatory Clinic | Jarab 2012[33] | -8.90 | 0.09 | -9.08 | -8.72 | -94.93 | 0.00 | 77 | 79 | 3.1 |
| Ambulatory Clinic | Korcegez 2017[35] | -3.40 | 0.46 | -4.29 | -2.51 | -7.45 | 0.00 | 75 | 77 | 3.1 |
| Ambulatory Clinic | Morgado 2011[39] | -1.90 | 0.36 | -2.61 | -1.19 | -5.26 | 0.00 | 76 | 99 | 3.1 |
| Ambulatory Clinic | Mourao 2013[40] | -0.50 | 0.12 | -0.73 | -0.27 | -4.22 | 0.00 | 50 | 50 | 3.1 |
| Ambulatory Clinic | Obreli-Neto 2011[42] | -12.90 | 0.16 | -13.22 | -12.58 | -78.66 | 0.00 | 97 | 97 | 3.1 |
| Ambulatory Clinic | Okamoto 2001[43] | -3.70 | 0.22 | -4.13 | -3.27 | -16.71 | 0.00 | 164 | 166 | 3.1 |
| Ambulatory Clinic | Plaster 2012[49] | -3.00 | 0.34 | -3.67 | -2.33 | -8.74 | 0.00 | 34 | 29 | 3.1 |
| Ambulatory Clinic | Polgreen 2015[50] | -2.50 | 0.12 | -2.74 | -2.26 | -20.04 | 0.00 | 401 | 224 | 3.1 |
| Ambulatory Clinic | Rothman 2005[52] | -9.00 | 0.13 | -9.25 | -8.75 | -70.00 | 0.00 | 112 | 105 | 3.1 |
| Ambulatory Clinic | Sanchez-guerra 2018[53] | -1.10 | 0.26 | -1.60 | -0.60 | -4.27 | 0.00 | 31 | 29 | 3.1 |
| Ambulatory Clinic | Shao 2017[55] | -3.40 | 0.39 | -4.16 | -2.64 | -8.80 | 0.00 | 100 | 99 | 3.1 |
| Ambulatory Clinic | Simpson 2011[56] | -2.90 | 0.12 | -3.13 | -2.67 | -25.14 | 0.00 | 129 | 131 | 3.1 |
| Ambulatory Clinic | Sookaneknun 2004[58] | -2.30 | 0.28 | -2.86 | -1.74 | -8.10 | 0.00 | 118 | 117 | 3.1 |
| Ambulatory Clinic | Tobari 2010[63] | -0.80 | 0.05 | -0.90 | -0.70 | -15.36 | 0.00 | 66 | 66 | 3.1 |
| Ambulatory Clinic | Wal 2013[67] | -5.10 | 0.41 | -5.90 | -4.30 | -12.47 | 0.00 | 54 | 48 | 3.1 |
| Ambulatory Clinic | | -4.53 | 0.62 | -5.75 | -3.32 | -7.29 | 0.00 | 3373 | 3246 | |
| Community Pharmacy | Amariles 2012[5] | -2.40 | 0.13 | -2.65 | -2.15 | -18.69 | 0.00 | 356 | 358 | 6.0 |
| Community Pharmacy | Bajorek 2016[7] | -8.00 | 0.58 | -9.14 | -6.86 | -13.77 | 0.00 | 10 | 11 | 5.6 |
| Community Pharmacy | Basheti 2016[8] | -6.60 | 0.06 | -6.72 | -6.48 | -111.04 | 0.00 | 82 | 78 | 6.1 |
| Community Pharmacy | Fornos 2006[24] | -2.00 | 0.27 | -2.53 | -1.47 | -7.40 | 0.00 | 56 | 56 | 6.0 |
| Community Pharmacy | Garcao 2002[25] | -7.10 | 0.79 | -8.65 | -5.55 | -8.96 | 0.00 | 41 | 41 | 5.3 |
| Community Pharmacy | Jahangard-Rafsanjani 2014[31] | -1.80 | 0.11 | -2.02 | -1.58 | -15.93 | 0.00 | 45 | 40 | 6.1 |
| Community Pharmacy | Krass 2007[36] | -1.40 | 0.06 | -1.51 | -1.29 | -24.54 | 0.00 | 87 | 92 | 6.1 |
| Community Pharmacy | Lugo De Ortellado 2008[38] | -4.40 | 0.21 | -4.81 | -3.99 | -20.80 | 0.00 | 33 | 28 | 6.0 |
| Community Pharmacy | Park 1996[45] | -5.00 | 0.64 | -6.25 | -3.75 | -7.87 | 0.00 | 23 | 26 | 5.5 |
| Community Pharmacy | Paulo 2016[46] | -0.50 | 0.17 | -0.84 | -0.16 | -2.88 | 0.00 | 47 | 42 | 6.0 |
| Community Pharmacy | Robinson 2010[51] | -1.90 | 0.10 | -2.09 | -1.71 | -19.38 | 0.00 | 78 | 62 | 6.1 |
| Community Pharmacy | Skowron 2010[57] | -1.00 | 0.36 | -1.70 | -0.30 | -2.79 | 0.01 | 28 | 56 | 5.9 |
| Community Pharmacy | Stewart 2014[59] | -0.30 | 0.22 | -0.73 | 0.13 | -1.36 | 0.17 | 176 | 176 | 6.0 |
| Community Pharmacy | Torres 2009[64] | -2.50 | 0.06 | -2.62 | -2.38 | -41.28 | 0.00 | 183 | 183 | 6.1 |
| Community Pharmacy | Villeneuve 2010[66] | -2.00 | 0.05 | -2.11 | -1.89 | -37.30 | 0.00 | 108 | 117 | 6.1 |
| Community Pharmacy | Wang 2011[68] | -4.10 | 0.80 | -5.67 | -2.53 | -5.12 | 0.00 | 29 | 30 | 5.3 |
| Community Pharmacy | Zillich 2005[70] | -3.20 | 0.15 | -3.50 | -2.90 | -20.65 | 0.00 | 64 | 61 | 6.0 |
| Community Pharmacy | | -3.13 | 0.50 | -4.11 | -2.14 | -6.23 | 0.00 | 1446 | 1457 | |
| Overall | | -3.68 | 0.39 | -4.45 | -2.92 | -9.43 | 0.00 | 4819 | 4703 | |

**Difference in means and 95% CI**

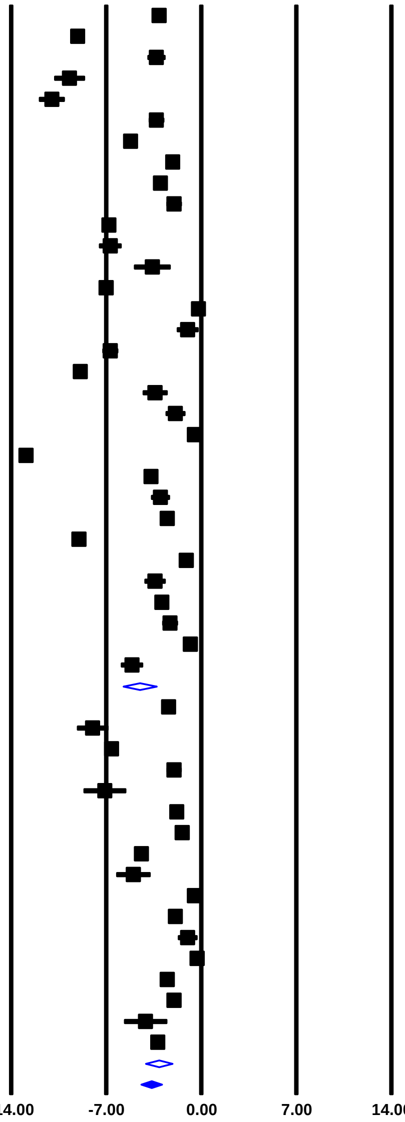


CI: confidence interval.

**Figure S3**. **Raw mean difference on glycated hemoglobin (HbA1C) in percentage.**

# Mean difference in HbA1c

| Group by Setting | Study name | Difference in means | Standard error | Lower limit | Upper limit | Z-Value | p-Value | Med Review | Usual Care | Relative weight |
|---|---|---|---|---|---|---|---|---|---|---|
| | | Statistics for each study | | | | | | Sample size | | |
| Ambulatory Clinic | Abuloha 2016[1] | -0.90 | 0.12 | -1.13 | -0.67 | -7.73 | 0.00 | 45 | 43 | 5.4 |
| Ambulatory Clinic | Aguiar 2016[2] | -0.70 | 0.01 | -0.73 | -0.67 | -49.47 | 0.00 | 36 | 37 | 5.7 |
| Ambulatory Clinic | Al Mazroui 2009[3] | -1.50 | 0.01 | -1.52 | -1.48 | -134.19 | 0.00 | 117 | 117 | 5.7 |
| Ambulatory Clinic | Chan 2012[14] | -1.20 | 0.14 | -1.46 | -0.94 | -8.88 | 0.00 | 51 | 54 | 5.3 |
| Ambulatory Clinic | Chen 2016[15] | -1.40 | 0.07 | -1.54 | -1.26 | -19.47 | 0.00 | 50 | 50 | 5.6 |
| Ambulatory Clinic | Choe 2005[16] | -1.20 | 0.10 | -1.40 | -1.00 | -11.80 | 0.00 | 36 | 29 | 5.4 |
| Ambulatory Clinic | Clifford 2002[18] | -0.20 | 0.03 | -0.25 | -0.15 | -7.69 | 0.00 | 48 | 25 | 5.6 |
| Ambulatory Clinic | Clifford 2005[19] | -0.50 | 0.01 | -0.52 | -0.48 | -40.69 | 0.00 | 92 | 88 | 5.7 |
| Ambulatory Clinic | Jacobs 2012[30] | -1.00 | 0.07 | -1.15 | -0.85 | -13.46 | 0.00 | 72 | 92 | 5.5 |
| Ambulatory Clinic | Jameson 2010[32] | -1.10 | 0.03 | -1.17 | -1.03 | -32.93 | 0.00 | 52 | 51 | 5.6 |
| Ambulatory Clinic | Jarab 2012[33] | -0.90 | 0.03 | -0.96 | -0.84 | -28.85 | 0.00 | 77 | 79 | 5.6 |
| Ambulatory Clinic | Korcegez 2017[35] | -0.70 | 0.04 | -0.78 | -0.62 | -16.82 | 0.00 | 75 | 77 | 5.6 |
| Ambulatory Clinic | Mourao 2013[40] | -1.40 | 0.02 | -1.44 | -1.36 | -62.76 | 0.00 | 50 | 50 | 5.6 |
| Ambulatory Clinic | Obreli-Neto 2011[42] | -0.70 | 0.03 | -0.76 | -0.64 | -24.28 | 0.00 | 97 | 97 | 5.6 |
| Ambulatory Clinic | Rothman 2005[52] | -0.90 | 0.02 | -0.94 | -0.86 | -41.87 | 0.00 | 99 | 95 | 5.7 |
| Ambulatory Clinic | Shao 2017[55] | -0.80 | 0.15 | -1.10 | -0.50 | -5.20 | 0.00 | 100 | 99 | 5.2 |
| Ambulatory Clinic | Simpson 2011[56] | -0.30 | 0.01 | -0.32 | -0.28 | -37.07 | 0.00 | 131 | 129 | 5.7 |
| Ambulatory Clinic | Wishah 2014[69] | -1.40 | 0.10 | -1.59 | -1.21 | -14.60 | 0.00 | 52 | 54 | 5.5 |
| Ambulatory Clinic | | -0.93 | 0.12 | -1.17 | -0.69 | -7.63 | 0.00 | 1280 | 1266 | |
| Community Pharmacy | Chung 2014[17] | -1.20 | 0.04 | -1.27 | -1.13 | -32.03 | 0.00 | 120 | 121 | 15.1 |
| Community Pharmacy | Doucette 2009[21] | -0.40 | 0.21 | -0.80 | 0.00 | -1.94 | 0.05 | 36 | 42 | 10.8 |
| Community Pharmacy | Fornos 2006[24] | -1.20 | 0.03 | -1.27 | -1.13 | -35.90 | 0.00 | 56 | 56 | 15.2 |
| Community Pharmacy | Jahangard-Rafsanjani 2014[31] | -0.50 | 0.04 | -0.57 | -0.43 | -13.36 | 0.00 | 45 | 40 | 15.1 |
| Community Pharmacy | Krass 2007[36] | -0.70 | 0.02 | -0.74 | -0.66 | -30.99 | 0.00 | 125 | 107 | 15.3 |
| Community Pharmacy | Paulo 2016[46] | -0.20 | 0.13 | -0.46 | 0.06 | -1.52 | 0.13 | 47 | 42 | 13.1 |
| Community Pharmacy | Taylor 2005[62] | -0.50 | 0.01 | -0.52 | -0.48 | -45.80 | 0.00 | 53 | 46 | 15.3 |
| Community Pharmacy | | -0.69 | 0.12 | -0.94 | -0.45 | -5.55 | 0.00 | 482 | 454 | |
| Overall | | -0.81 | 0.09 | -0.99 | -0.64 | -9.33 | 0.00 | 1762 | 1720 | |

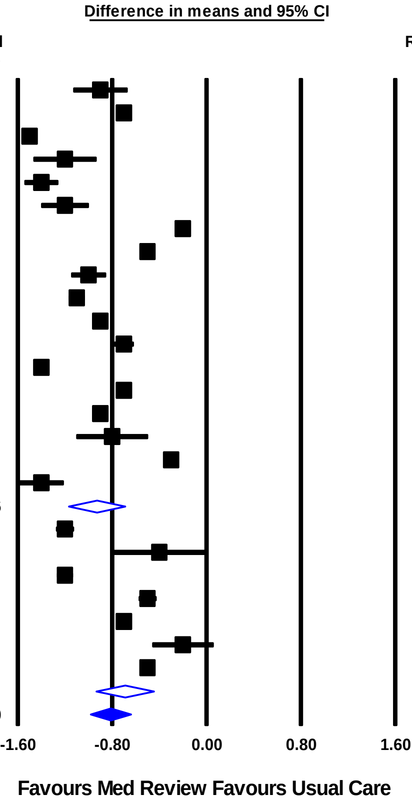


CI: confidence interval.

**Figure S4. Raw mean difference on fasting glucose in milligrams per decilitre.**

# Mean difference in Fasting Glucose

| Group by Setting | Study name | Difference in means | Standard error | Lower limit | Upper limit | Z-Value | p-Value | Med Review | Usual Care | Relative weight |
|---|---|---|---|---|---|---|---|---|---|---|
| | | **Statistics for each study** | | | | | | **Sample size** | | |
| Ambulatory Clinic | Abuloha 2016[1] | -42.0 | 1.78 | -45.5 | -38.5 | -23.58 | 0.00 | 45 | 43 | 7.7 |
| Ambulatory Clinic | Al Mazroui 2009[3] | -40.8 | 2.43 | -45.6 | -36.0 | -16.76 | 0.00 | 117 | 117 | 7.7 |
| Ambulatory Clinic | Azevedo 2017[6] | -22.4 | 4.24 | -30.7 | -14.1 | -5.29 | 0.00 | 33 | 30 | 7.4 |
| Ambulatory Clinic | Clifford 2005[19] | -21.7 | 0.29 | -22.3 | -21.1 | -73.99 | 0.00 | 92 | 88 | 7.8 |
| Ambulatory Clinic | Firminho 2015[23] | -7.2 | 2.60 | -12.3 | -2.1 | -2.77 | 0.01 | 26 | 30 | 7.7 |
| Ambulatory Clinic | Hammad 2011[27] | -3.3 | 0.37 | -4.0 | -2.6 | -8.83 | 0.00 | 110 | 89 | 7.8 |
| Ambulatory Clinic | Jarab 2012[33] | -57.6 | 0.74 | -59.1 | -56.1 | -77.41 | 0.00 | 77 | 79 | 7.8 |
| Ambulatory Clinic | Korcegez 2017[35] | -7.7 | 2.01 | -11.6 | -3.8 | -3.82 | 0.00 | 75 | 77 | 7.7 |
| Ambulatory Clinic | Mourao 2013[40] | -34.8 | 0.57 | -35.9 | -33.7 | -60.76 | 0.00 | 50 | 50 | 7.8 |
| Ambulatory Clinic | Obreli-Neto 2011[42] | -28.3 | 0.36 | -29.0 | -27.6 | -79.35 | 0.00 | 97 | 97 | 7.8 |
| Ambulatory Clinic | Plaster 2012[49] | -60.0 | 0.60 | -61.2 | -58.8 | -99.51 | 0.00 | 34 | 29 | 7.8 |
| Ambulatory Clinic | Shao 2017[55] | -24.5 | 2.63 | -29.7 | -19.3 | -9.31 | 0.00 | 100 | 99 | 7.7 |
| Ambulatory Clinic | Wishah 2014[69] | -51.7 | 4.74 | -61.0 | -42.4 | -10.91 | 0.00 | 52 | 54 | 7.3 |
| Ambulatory Clinic | | -30.9 | 5.15 | -41.0 | -20.8 | -6.00 | 0.00 | 908 | 882 | |
| Community Pharmacy | Basheti 2016[8] | -19.5 | 2.37 | -24.2 | -14.8 | -8.22 | 0.00 | 82 | 78 | 25.2 |
| Community Pharmacy | Chung 2014[17] | -43.3 | 1.95 | -47.1 | -39.5 | -22.21 | 0.00 | 120 | 121 | 25.3 |
| Community Pharmacy | Paulo 2016[46] | -7.9 | 5.80 | -19.3 | 3.5 | -1.36 | 0.17 | 47 | 42 | 24.0 |
| Community Pharmacy | Villeneuve 2010[66] | -2.0 | 0.32 | -2.6 | -1.4 | -6.34 | 0.00 | 108 | 117 | 25.5 |
| Community Pharmacy | | -18.3 | 11.62 | -41.1 | 4.5 | -1.57 | 0.12 | 357 | 358 | |
| Overall | | -28.8 | 4.71 | -38.1 | -19.6 | -6.12 | 0.00 | 1265 | 1240 | |

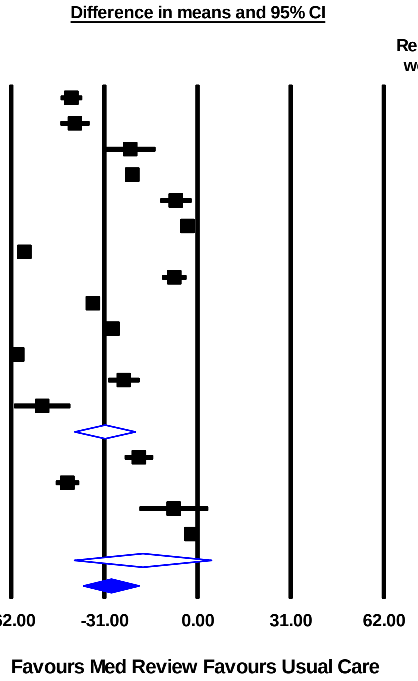


CI: confidence interval.

**Figure S5. Raw mean difference on total cholesterol in milligrams per decilitre.**

## Mean difference in Total Cholesterol

| Group by Setting | Study name | Difference in means | Standard error | Lower limit | Upper limit | Z-Value | p-Value | Med Review | Usual Care | Relative weight |
|---|---|---|---|---|---|---|---|---|---|---|
| | | Statistics for each study | | | | | | Sample size | | |
| Ambulatory Clinic | Al Mazroui 2009 [3] | -32.4 | 1.09 | -34.5 | -30.3 | -29.82 | 0.00 | 117 | 117 | 5.9 |
| Ambulatory Clinic | Azevedo 2017 [6] | -12.0 | 1.92 | -15.8 | -8.2 | -6.23 | 0.00 | 33 | 33 | 5.8 |
| Ambulatory Clinic | Bogden 1997 [10] | -31.0 | 0.44 | -31.9 | -30.1 | -70.55 | 0.00 | 47 | 47 | 6.0 |
| Ambulatory Clinic | Chan 2012 [14] | -12.3 | 0.33 | -13.0 | -11.6 | -36.72 | 0.00 | 51 | 54 | 6.0 |
| Ambulatory Clinic | Clifford 2005 [19] | -3.8 | 0.25 | -4.3 | -3.3 | -15.06 | 0.00 | 92 | 88 | 6.0 |
| Ambulatory Clinic | Firminho 2015 [23] | -13.7 | 2.13 | -17.9 | -9.5 | -6.43 | 0.00 | 21 | 15 | 5.7 |
| Ambulatory Clinic | Jarab 2012 [33] | -31.2 | 0.41 | -32.0 | -30.4 | -76.94 | 0.00 | 77 | 79 | 6.0 |
| Ambulatory Clinic | Korcegez 2017 [35] | -6.8 | 1.24 | -9.2 | -4.4 | -5.47 | 0.00 | 77 | 75 | 5.9 |
| Ambulatory Clinic | Lee 2009 [37] | -23.6 | 1.48 | -26.5 | -20.7 | -15.95 | 0.00 | 58 | 60 | 5.9 |
| Ambulatory Clinic | Mourao 2013 [40] | -25.6 | 0.55 | -26.7 | -24.5 | -46.55 | 0.00 | 50 | 50 | 5.9 |
| Ambulatory Clinic | Obreli-Neto 2011 [42] | -21.0 | 2.32 | -25.6 | -16.4 | -9.04 | 0.00 | 97 | 97 | 5.7 |
| Ambulatory Clinic | Plaster 2012 [49] | -24.0 | 0.91 | -25.8 | -22.2 | -26.48 | 0.00 | 34 | 29 | 5.9 |
| Ambulatory Clinic | Rothman 2005 [52] | -15.0 | 0.45 | -15.9 | -14.1 | -33.63 | 0.00 | 99 | 95 | 6.0 |
| Ambulatory Clinic | Shao 2017 [55] | -19.0 | 0.35 | -19.7 | -18.3 | -54.51 | 0.00 | 100 | 99 | 6.0 |
| Ambulatory Clinic | Simpson 2011 [56] | -5.4 | 0.21 | -5.8 | -5.0 | -25.38 | 0.00 | 131 | 129 | 6.0 |
| Ambulatory Clinic | Villa 2009 [65] | -22.0 | 0.90 | -23.8 | -20.2 | -24.47 | 0.00 | 85 | 57 | 5.9 |
| Ambulatory Clinic | Wishah 2014 [69] | -7.5 | 2.42 | -12.2 | -2.8 | -3.10 | 0.00 | 52 | 54 | 5.7 |
| Ambulatory Clinic | | -18.0 | 2.62 | -23.2 | -12.9 | -6.88 | 0.00 | 1221 | 1178 | |
| Community Pharmacy | Amariles 2012 [5] | -4.6 | 0.83 | -6.2 | -3.0 | -5.56 | 0.00 | 356 | 358 | 14.7 |
| Community Pharmacy | Fornos 2006 [24] | -19.0 | 0.73 | -20.4 | -17.6 | -26.18 | 0.00 | 56 | 56 | 14.7 |
| Community Pharmacy | Krass 2007 [36] | 0.0 | 0.25 | -0.5 | 0.5 | 0.00 | 1.00 | 112 | 98 | 14.8 |
| Community Pharmacy | Nola 2000 [41] | -5.6 | 2.21 | -9.9 | -1.3 | -2.53 | 0.01 | 25 | 26 | 13.7 |
| Community Pharmacy | Paulo 2016 [46] | -10.0 | 0.39 | -10.8 | -9.2 | -25.70 | 0.00 | 47 | 42 | 14.8 |
| Community Pharmacy | Paulos 2005 [47] | -22.9 | 3.23 | -29.2 | -16.6 | -7.09 | 0.00 | 23 | 19 | 12.6 |
| Community Pharmacy | Villeneuve 2010 [66] | -7.7 | 0.81 | -9.3 | -6.1 | -9.55 | 0.00 | 108 | 117 | 14.7 |
| Community Pharmacy | | -9.7 | 2.92 | -15.5 | -4.0 | -3.33 | 0.00 | 727 | 716 | |
| Overall | | -14.3 | 1.95 | -18.2 | -10.5 | -7.34 | 0.00 | 1948 | 1894 | |

Difference in means and 95% CI

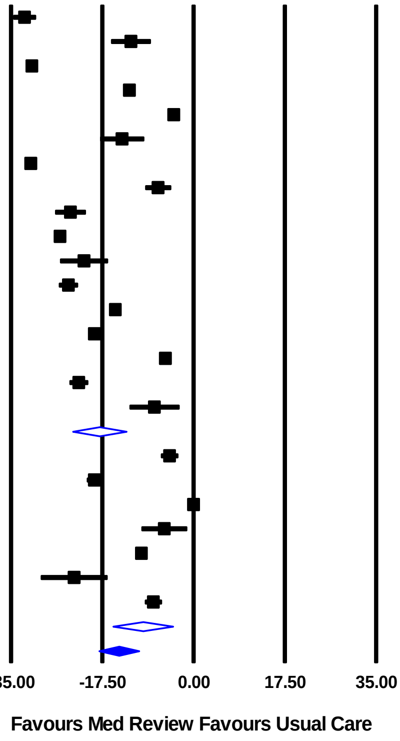


CI: confidence interval.

**Figure S6. Raw mean difference on Low Density Lipoprotein (LDL) cholesterol in milligrams per decilitre.**

## Mean difference in LDL Cholesterol

| Group by Setting | Study name | Difference in means | Standard error | Lower limit | Upper limit | Z-Value | p-Value | Med Review | Usual Care | Relative weight |
|---|---|---|---|---|---|---|---|---|---|---|
| Ambulatory Clinic | Aguiar 2016 [2] | 0.0 | 0.369 | -0.7 | 0.7 | 0.00 | 1.00 | 36 | 37 | 6.8 |
| Ambulatory Clinic | Al Mazroui 2009 [3] | -29.0 | 1.276 | -31.5 | -26.5 | -22.74 | 0.00 | 117 | 117 | 6.6 |
| Ambulatory Clinic | Azevedo 2017 [6] | -14.0 | 0.611 | -15.2 | -12.8 | -22.92 | 0.00 | 33 | 33 | 6.8 |
| Ambulatory Clinic | Chan 2012 [14] | -12.4 | 0.270 | -12.9 | -11.9 | -45.98 | 0.00 | 51 | 54 | 6.8 |
| Ambulatory Clinic | Firminho 2015 [23] | -6.0 | 3.221 | -12.3 | 0.3 | -1.86 | 0.06 | 21 | 15 | 5.7 |
| Ambulatory Clinic | Jarab 2012 [33] | -23.2 | 0.444 | -24.1 | -22.3 | -52.21 | 0.00 | 77 | 79 | 6.8 |
| Ambulatory Clinic | Korcegez 2017 [35] | -6.6 | 1.335 | -9.2 | -4.0 | -4.94 | 0.00 | 77 | 75 | 6.6 |
| Ambulatory Clinic | Lee 2009 [37] | -22.8 | 1.184 | -25.1 | -20.5 | -19.25 | 0.00 | 58 | 60 | 6.7 |
| Ambulatory Clinic | Mourao 2013 [40] | -24.5 | 0.480 | -25.4 | -23.6 | -51.01 | 0.00 | 50 | 50 | 6.8 |
| Ambulatory Clinic | Obreli-Neto 2011 [42] | -13.9 | 0.083 | -14.1 | -13.7 | -167.86 | 0.00 | 97 | 97 | 6.9 |
| Ambulatory Clinic | Plaster 2012 [49] | -27.0 | 0.394 | -27.8 | -26.2 | -68.50 | 0.00 | 34 | 29 | 6.8 |
| Ambulatory Clinic | Shao 2017 [55] | -11.2 | 0.413 | -12.0 | -10.4 | -27.13 | 0.00 | 100 | 99 | 6.8 |
| Ambulatory Clinic | Simpson 2011 [56] | -5.0 | 0.168 | -5.3 | -4.7 | -29.77 | 0.00 | 131 | 129 | 6.9 |
| Ambulatory Clinic | Villa 2009 [65] | -23.0 | 0.488 | -24.0 | -22.0 | -47.15 | 0.00 | 85 | 57 | 6.8 |
| Ambulatory Clinic | Wishah 2014 [69] | -9.3 | 2.530 | -14.3 | -4.3 | -3.68 | 0.00 | 52 | 54 | 6.1 |
| Ambulatory Clinic | | -15.3 | 1.843 | -18.9 | -11.7 | -8.31 | 0.00 | 1019 | 985 | |
| Community Pharmacy | Doucette 2009 [21] | -7.6 | 0.335 | -8.3 | -6.9 | -22.72 | 0.00 | 36 | 42 | 21.1 |
| Community Pharmacy | Fornos 2006 [24] | -12.0 | 0.506 | -13.0 | -11.0 | -23.69 | 0.00 | 56 | 56 | 20.5 |
| Community Pharmacy | Nola 2000 [41] | -10.4 | 1.309 | -13.0 | -7.8 | -7.94 | 0.00 | 25 | 26 | 15.9 |
| Community Pharmacy | Paulo 2016 [46] | -5.7 | 0.318 | -6.3 | -5.1 | -17.91 | 0.00 | 47 | 42 | 21.2 |
| Community Pharmacy | Villeneuve 2010 [66] | -8.8 | 0.282 | -9.4 | -8.2 | -31.25 | 0.00 | 108 | 117 | 21.3 |
| Community Pharmacy | | -8.8 | 1.012 | -10.8 | -6.8 | -8.70 | 0.00 | 272 | 283 | |
| Overall | | -10.3 | 0.887 | -12.0 | -8.6 | -11.63 | 0.00 | 1291 | 1268 | |

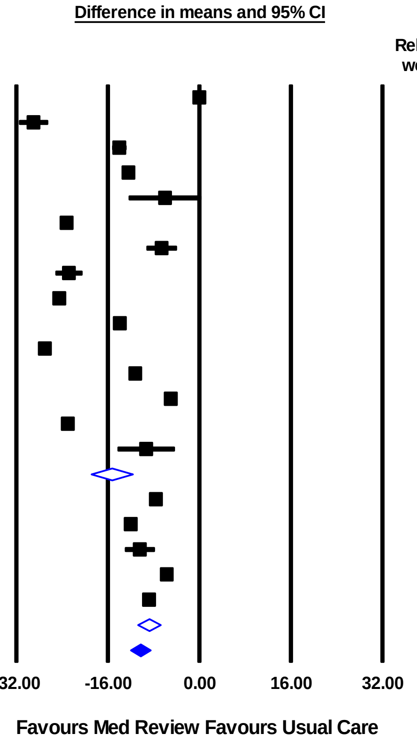


CI: confidence interval.

**Figure S7**. Raw mean difference on High Density Lipoprotein (HDL) cholesterol in milligrams per decilitre.

# Mean difference in HDL Cholesterol

| Group by Setting | Study name | Difference in means | Standard error | Lower limit | Upper limit | Z-Value | p-Value | Med Review | Usual Care | Relative weight |
|---|---|---|---|---|---|---|---|---|---|---|
| | | Statistics for each study | | | | | | Sample size | | |
| Ambulatory Clinic | Al Mazroui 2009[3] | 4.30 | 0.20 | 3.90 | 4.70 | 21.12 | 0.00 | 117 | 117 | 6.3 |
| Ambulatory Clinic | Azevedo 2017[6] | 8.50 | 0.17 | 8.18 | 8.82 | 51.50 | 0.00 | 33 | 30 | 6.3 |
| Ambulatory Clinic | Chan 2012[14] | 0.10 | 0.09 | -0.07 | 0.27 | 1.16 | 0.25 | 51 | 54 | 6.3 |
| Ambulatory Clinic | Clifford 2005[19] | 2.00 | 0.09 | 1.82 | 2.18 | 21.24 | 0.00 | 92 | 88 | 6.3 |
| Ambulatory Clinic | Firminho 2015[23] | 3.30 | 0.66 | 2.01 | 4.59 | 5.01 | 0.00 | 21 | 15 | 6.2 |
| Ambulatory Clinic | Hammad 2011[27] | 5.00 | 0.13 | 4.74 | 5.26 | 37.21 | 0.00 | 110 | 89 | 6.3 |
| Ambulatory Clinic | Jarab 2012[33] | 5.80 | 0.23 | 5.36 | 6.24 | 25.75 | 0.00 | 77 | 79 | 6.3 |
| Ambulatory Clinic | Korcegez 2017[35] | 0.00 | 0.11 | -0.22 | 0.22 | 0.00 | 1.00 | 77 | 75 | 6.3 |
| Ambulatory Clinic | Lee 2009[37] | 0.70 | 0.40 | -0.08 | 1.48 | 1.76 | 0.08 | 58 | 60 | 6.2 |
| Ambulatory Clinic | Mourao 2013[40] | 4.50 | 0.12 | 4.27 | 4.73 | 38.95 | 0.00 | 50 | 50 | 6.3 |
| Ambulatory Clinic | Obreli-Neto 2011[42] | 10.00 | 0.05 | 9.90 | 10.10 | 196.49 | 0.00 | 97 | 97 | 6.3 |
| Ambulatory Clinic | Plaster 2012[49] | 11.30 | 0.04 | 11.23 | 11.37 | 314.82 | 0.00 | 34 | 29 | 6.3 |
| Ambulatory Clinic | Shao 2017[55] | 4.20 | 0.32 | 3.58 | 4.82 | 13.28 | 0.00 | 100 | 99 | 6.2 |
| Ambulatory Clinic | Simpson 2011[56] | 0.20 | 0.28 | -0.34 | 0.74 | 0.73 | 0.47 | 131 | 129 | 6.3 |
| Ambulatory Clinic | Villa 2009[65] | 3.00 | 0.51 | 2.00 | 4.00 | 5.85 | 0.00 | 85 | 57 | 6.2 |
| Ambulatory Clinic | Wishah 2014[69] | 2.20 | 0.18 | 1.86 | 2.54 | 12.51 | 0.00 | 52 | 54 | 6.3 |
| Ambulatory Clinic | | 4.07 | 1.23 | 1.66 | 6.49 | 3.31 | 0.00 | 1185 | 1122 | |
| Community Pharmacy | Fornos 2006[24] | 0.20 | 0.12 | -0.03 | 0.43 | 1.67 | 0.09 | 56 | 56 | 30.1 |
| Community Pharmacy | Nola 2000[41] | 3.80 | 0.80 | 2.23 | 5.37 | 4.73 | 0.00 | 25 | 26 | 7.8 |
| Community Pharmacy | Paulo 2016[46] | 0.40 | 0.11 | 0.19 | 0.61 | 3.79 | 0.00 | 47 | 42 | 30.6 |
| Community Pharmacy | Villeneuve 2010[66] | 0.90 | 0.07 | 0.76 | 1.04 | 12.91 | 0.00 | 108 | 117 | 31.5 |
| Community Pharmacy | | 0.76 | 0.26 | 0.26 | 1.27 | 2.95 | 0.00 | 236 | 241 | |
| Overall | | 0.90 | 0.25 | 0.41 | 1.40 | 3.57 | 0.00 | 1421 | 1363 | |

Difference in means and 95% CI

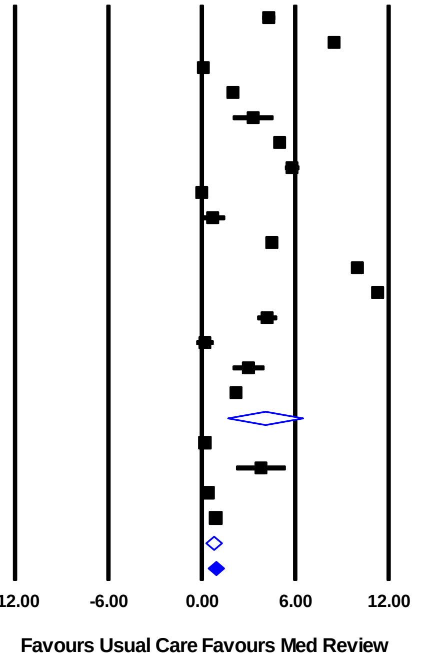


CI: confidence interval.

**Figure S8**. **Raw mean difference on Triglycerides in milligrams per decilitre.**

# Mean difference in Triglycerides

| Group by Setting | Study name | Statistics for each study: Difference in means | Standard error | Lower limit | Upper limit | Z-Value | p-Value | Sample size: Med Review | Usual Care | Relative weight |
|---|---|---|---|---|---|---|---|---|---|---|
| Ambulatory Clinic | Al Mazroui 2009 [3] | -47.8 | 2.72 | -53.1 | -42.5 | -17.58 | 0.00 | 117 | 117 | 6.4 |
| Ambulatory Clinic | Azevedo 2017 [6] | -63.9 | 9.16 | -81.9 | -45.9 | -6.97 | 0.00 | 33 | 30 | 5.2 |
| Ambulatory Clinic | Chan 2012 [14] | 0.0 | 6.25 | -12.2 | 12.2 | 0.00 | 1.00 | 51 | 54 | 5.9 |
| Ambulatory Clinic | Clifford 2005 [19] | -51.1 | 5.14 | -61.2 | -41.0 | -9.93 | 0.00 | 92 | 88 | 6.1 |
| Ambulatory Clinic | Firminho 2015 [23] | -52.4 | 3.16 | -58.6 | -46.2 | -16.60 | 0.00 | 21 | 15 | 6.4 |
| Ambulatory Clinic | Hammad 2011 [27] | -16.9 | 0.59 | -18.1 | -15.7 | -28.60 | 0.00 | 110 | 89 | 6.6 |
| Ambulatory Clinic | Jarab 2012 [33] | -62.1 | 1.58 | -65.2 | -59.0 | -39.26 | 0.00 | 77 | 79 | 6.5 |
| Ambulatory Clinic | Korcegez 2017 [35] | -1.2 | 0.64 | -2.4 | 0.0 | -1.88 | 0.06 | 77 | 75 | 6.6 |
| Ambulatory Clinic | Lee 2009 [37] | -15.0 | 1.45 | -17.8 | -12.2 | -10.33 | 0.00 | 58 | 60 | 6.5 |
| Ambulatory Clinic | Mourao 2013 [40] | -34.0 | 0.84 | -35.6 | -32.4 | -40.49 | 0.00 | 50 | 50 | 6.6 |
| Ambulatory Clinic | Obreli-Neto 2011 [42] | -51.6 | 0.87 | -53.3 | -49.9 | -59.48 | 0.00 | 97 | 97 | 6.6 |
| Ambulatory Clinic | Plaster 2012 [49] | -12.0 | 1.11 | -14.2 | -9.8 | -10.85 | 0.00 | 34 | 29 | 6.5 |
| Ambulatory Clinic | Shao 2017 [55] | -25.7 | 5.37 | -36.2 | -15.2 | -4.78 | 0.00 | 100 | 99 | 6.0 |
| Ambulatory Clinic | Simpson 2011 [56] | -16.0 | 0.50 | -17.0 | -15.0 | -31.85 | 0.00 | 131 | 129 | 6.6 |
| Ambulatory Clinic | Villa 2009 [65] | -77.0 | 4.89 | -86.6 | -67.4 | -15.74 | 0.00 | 85 | 57 | 6.1 |
| Ambulatory Clinic | Wishah 2014 [69] | -37.0 | 7.73 | -52.1 | -21.9 | -4.79 | 0.00 | 52 | 54 | 5.5 |
| Ambulatory Clinic | | -34.8 | 4.59 | -43.8 | -25.8 | -7.59 | 0.00 | 1185 | 1122 | |
| Community Pharmacy | Basheti 2016 [8] | -31.9 | 6.74 | -45.1 | -18.7 | -4.73 | 0.00 | 82 | 78 | 13.2 |
| Community Pharmacy | Fornos 2006 [24] | -32.0 | 7.04 | -45.8 | -18.2 | -4.55 | 0.00 | 56 | 56 | 13.0 |
| Community Pharmacy | Krass 2007 [36] | -17.7 | 0.52 | -18.7 | -16.7 | -33.97 | 0.00 | 112 | 97 | 17.1 |
| Community Pharmacy | Nola 2000 [41] | -2.8 | 8.15 | -18.8 | 13.2 | -0.34 | 0.73 | 25 | 26 | 12.0 |
| Community Pharmacy | Paulo 2016 [46] | -18.5 | 1.19 | -20.8 | -16.2 | -15.56 | 0.00 | 47 | 42 | 17.0 |
| Community Pharmacy | Paulos 2005 [47] | -80.0 | 9.69 | -99.0 | -61.0 | -8.26 | 0.00 | 23 | 19 | 10.6 |
| Community Pharmacy | Villeneuve 2010 [66] | 0.0 | 0.45 | -0.9 | 0.9 | 0.00 | 1.00 | 108 | 117 | 17.1 |
| Community Pharmacy | | -23.4 | 5.12 | -33.4 | -13.3 | -4.57 | 0.00 | 453 | 435 | |
| Overall | | -29.7 | 3.42 | -36.4 | -23.0 | -8.70 | 0.00 | 1638 | 1557 | |

Difference in means and 95% CI

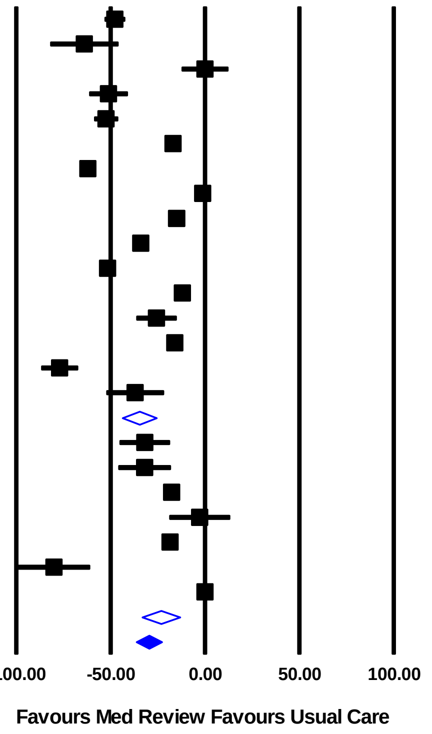


CI: confidence interval.

**Supplemental References:**


1. Abuloha S, Alabbadi I, Albsoul-Younes A, Younes N, Zayed A. The role of clinical pharmacist in initiation and/or dose adjustment of insulin therapy in diabetic patients in outpatient clinic in Jordan. JJPS. 2016; 9:33-50.
2. Aguiar PM, da Silva CHP, Chiann C, Dórea EL, Lyra DP, Storpirtis S. Pharmacist–physician collaborative care model for patients with uncontrolled type 2 diabetes in Brazil: results from a randomized controlled trial. J Eval Clin Pract. 2018; 24:22–30.
3. Al Mazroui NR, Kamal MM, Ghabash NM, Yacout TA, Kole PL, McElnay JC. Influence of pharmaceutical care on health outcomes in patients with Type 2 diabetes mellitus. Brit J Clin Pharmaco. 2009; 67:547–557.
4. Albsoul-Younes AM, Hammad EA, Yasein NA, Tahaineh LM. Pharmacist-physician collaboration improves blood pressure control. Saudi Med J. 2011; 32:288–292.
5. Amariles P, Sabater-Hernández D, García-Jiménez E, Rodríguez-Chamorro MA, Prats-Más R, Marín-Magán F, Galán-Ceballos JA, Jiménez-Martín J, Faus MJ. Effectiveness of Dader method for pharmaceutical care on control of blood pressure and total cholesterol in outpatients with cardiovascular disease or cardiovascular risk: EMDADER-CV randomized controlled trial. J Manag Care Pharm. 2012; 18:311–23.
6. Azevedo, MGB, Pedrosa RS, Aoqui CM, Martins RR, Junior TN. Effectiveness of home pharmaceutical interventions in metabolic syndrome: a randomized controlled trial. Braz J Pharm Sci. 2017; 53:e16089.
7. Bajorek B, Lemay, KS, Magin P, Roberts C, Krass I, Armour CL. Implementation and evaluation of a pharmacist-led hypertension management service in primary care: outcomes and methodological challenges. Pharmacy Pract. 2016; 14: 723.
8. Basheti IA, Tadros OK, Aburuz S. Value of a Community-Based Medication Management Review Service in Jordan: A Prospective Randomized Controlled Study. Pharmacotherapy. 2016; 36:1075-1086.
9. Bogden PE, Abbott RD, Williamson P, Onopa JK, Koontz LM. Comparing standard care with a physician and pharmacist team approach for uncontrolled hypertension. J Gen Intern Med. 1998; 13:740-745.
10. Bogden PE, Koontz LM, Williamson P, Abbott RD. The physician and pharmacist team. An effective approach to cholesterol reduction. J Gen Intern Med. 1997; 12:158-164.
11. Borenstein JE, Graber G, Saltiel E, Wallace J, Ryu S, Jackson A, Deutsch S, Weingarten SR. Physician-Pharmacist Comanagement of Hypertension: A Randomized, Comparative Trial. Pharmacotherapy. 2003; 23:209-216.
12. Carter BL, Bergus GR, Dawson JD, Farris KB, Doucette WR, Chrischilles EA, Hartz AJ. A Cluster-Randomized Trial to Evaluate Physician/Pharmacist Collaboration to Improve Blood Pressure Control. J Clin Hypertens. 2008; 10:260–271.
13. Carter BL, Ardery G, Dawson JD, James PA, Bergus GR, Doucette WR, Chrischilles EA, Franciscus CL, Xu Y. Physician/Pharmacist Collaboration to Improve Blood Pressure Control. Arch Intern Med. 2009; 169:1996–2002.
14. Chan CW, Siu SC, Wong CK, Lee VW. A pharmacist care program: positive impact on cardiac risk in patients with type 2 diabetes. J Cardiovasc Pharmacol Ther. 2012; 17:57–64.

15. Chen JH, Ou HT, Lin TC, Lai EC, Kao YH. Pharmaceutical care of elderly patients with poorly controlled type 2 diabetes mellitus: a randomized controlled trial. Int J Clin Pharm. 2016; 38:88-95.
16. Choe HM, Mitrovich S, Dubay D, Hayward RA, Krein SL, Vijan S. Proactive case management of high-risk patients with type 2 diabetes mellitus by a clinical pharmacist: a randomised controlled trial. Am J Manag Care. 2005; 11:253–260.
17. Chung WW, Chua SS, Lai PSM, Chan SP. Effects of a pharmaceutical care model on medication adherence and glycemic control of people with type 2 diabetes. Patient Prefer Adher. 2014; 8:1185–1194.
18. Clifford RM, Batty KT, Davis TM, Davis W, Stein G, Stewart G, Plumridge RJ. A randomised controlled trial of a pharmaceutical care programme in high-risk diabetic patients in an outpatient clinic. Int J Pharm Pract. 2002; 10:85-89.
19. Clifford RM, Davis WA, Batty KT, Davis TM. Effect of a pharmaceutical care program on vascular risk factors in type 2 diabetes: the Fremantle Diabetes Study. Diabetes Care. 2005; 28:771-776
20. de Castro MS, Fuchs FD, Santos MC, Maximiliano P, Gus M, Moreira LB, Ferreira MB. Pharmaceutical care program for patients with uncontrolled hypertension. Report of a double-blind clinical trial with ambulatory blood pressure monitoring. Am J Hypertens. 2006; 19:528–533.
21. Doucette WR, Witry MJ, Farris KB, McDonough RP. Community pharmacist-provided extended diabetes care. Ann Pharmacother. 2009; 43:882–889.
22. Ebid AHIM, Ali ZT, Ghobary MAF. Blood pressure control in hypertensive patients: Impact of an Egyptian pharmaceutical care model. J App Pharm Sci. 2014; 4:093-101.
23. Firminho PYM, Vasconcelos TO, Ferreira CC, Moreira LM, Romero NR, Dias LA, de Queiroz MGR, Lopes MVO, Fonteles MMF. Cardiovascular risk rate in hypertensive patients attended in primary health care units: the influence of pharmaceutical care. Braz J Pharm Sci. 2015; 51:617-627
24. Fornos JA, Andres NF, Andres JC, Guerra MM, Egea B. A pharmacotherapy follow-up program in patients with type-2 diabetes in community pharmacies in Spain. Pharm World Sci. 2006; 28:65–72.
25. Garcao JA, Cabrita J. Evaluation of a pharmaceutical care program for hypertensive patients in rural Portugal. J Am Pharm Assoc. 2002; 42:858–864.
26. Green BB, Cook AJ, Ralston JD, Fishman PA, Catz SL, Carlson J, Thompson RS. Effectiveness of Home Blood Pressure Monitoring, Web Communication, and Pharmacist Care on Hypertension Control: The e-BP Randomized Controlled Trial. JAMA. 2008; 299:2857–2867.
27. Hammad EA, Yasein N, Tahaineh L, Albsoul-Younes AM. A randomized controlled trial to assess pharmacist-physician collaborative practice in the management of metabolic syndrome in a university medical clinic in Jordan. J Manag Care Pharm, 2011; 17:295-303.
28. Hedegaard U, Kjeldsen LJ, Pottegård A, Henriksen JE, Lambrechtsen J, Hangaard J, Hallas J. Improving Medication Adherence in Patients with Hypertension: A Randomized Trial. Am J Med. 2015; 128:1351-1361.
29. Hunt JS, Siemienczuk J, Pape G, Rozenfeld Y, Mackay J, LeBlanc BH, Touchette D. A Randomized Controlled Trial of Team-Based Care: Impact of Physician-Pharmacist Collaboration on Uncontrolled Hypertension. J Gen Intern Med. 2008; 23:1966-1972.

30. Jacobs M, Sherry PS, Taylor LM, Amato M, Tataronis GR, Cushing G. Pharmacist Assisted Medication Program Enhancing the Regulation of Diabetes (PAMPERED) study. J Am Pharm Assoc. 2012; 52:613–621.
31. Jahangard-Rafsanjani Z, Sarayani A, Nosrati M, Saadat N, Rashidian A, Hadjibabaie M, Ashouri A, Radfar M, Javadi M, Gholami K. Effect of a community pharmacist-delivered diabetes support program for patients receiving specialty medical care: a randomized controlled trial. Diabetes Educ. 2015; 41;127-135.
32. Jameson JP, Baty PJ. Pharmacist collaborative management of poorly controlled diabetes mellitus: a randomized controlled trial. Am J Manag Care. 2010; 16:250–255
33. Jarab AS, Alqudah SG, Mukattash TL, Shattat G, Al-Qirim T. Randomized controlled trial of clinical pharmacy management of patients with type 2 diabetes in an outpatient diabetes clinic in Jordan. J Manag Care Pharm. 2012; 18:516–526.
34. Kjeldsen LJ, Bjerrum L, Dam P, Larsen BO, Rossing C, Søndergaard B, Herborg H. Safe and effective use of medicines for patients with type 2 diabetes – a randomized controlled trial of two interventions delivered by local pharmacies. Res Social Adm Pharm. 2015; 11:47–62
35. Korcegez EI, Sancar M, Demirkan K. Effect of a pharmacist-led program on improving outcomes in patients with type 2 diabetes mellitus from Northern Cyprus: a randomized controlled trial. J Manag Care Spec Pharm. 2017; 23:573–582.
36. Krass, I, Armour, CL, Mitchell B, Brillant M, Dienaar R, Hughes J, Lau P, Peterson G, Stewart K, Taylor S, Wilkinson J. The Pharmacy Diabetes Care Program: assessment of a community pharmacy diabetes service model in Australia. Diabetic Medicine. 2007; 24:677-683.
37. Lee VW, Fan CS, Li AW, Chau AC. Clinical impact of a pharmacist-physician co-managed programme on hyperlipidaemia management in Hong Kong. J Clin Pharm Ther. 2009; 34:407–414.
38. Lugo De Ortellado G, De Bittner MR, Chavez GH, Perez S. Implementación de un programa de atención farmacéutica en farmacias comunitarias para la detección de la hipertensión arterial y su seguimiento farmacoterapéutico. Lat Am J Pharm. 2007; 4:590-595.
39. Morgado M, Rolo S, Castelo-Branco M. Pharmacist intervention program to enhance hypertension control: a randomised controlled trial. Int J Clin Pharm. 2011; 33:132-140.
40. Mourao AO, Ferreira WR, Martins MA, Reis AM, Carrillo MR, Guimaraes AG, Ev LS. Pharmaceutical care program for type 2 diabetes patients in Brazil: a randomised controlled trial. Int J Clin Pharm. 2013; 35:79-86
41. Nola KM, Gourley DR, Portner TS, Gourley GK, Solomon DK, Elam M, Regel B. Clinical and humanistic outcomes of a lipid management program in the community pharmacy setting. J Am Pharm Assoc. 2000; 40:166–173.
42. Obreli-Neto PR, Marusic S, de Lyra Junior DP, Pilger D, Cruciol-Souza JM, Gaeti WP, Cuman RK. Effect of a 36-month pharmaceutical care program on the coronary heart disease risk in elderly diabetic and hypertensive patients. J Pharm Pharmac Sci. 2011; 14:249-263.
43. Okamoto MP, Nakahiro RK. Pharmacoeconomic evaluation of a pharmacist-managed hypertension clinic. Pharmacotherapy. 2001; 21:1337–1344.
44. Oparah AC, Famakinde AJ, Adebaya OJ2. Outcomes of pharmacists' interventions in the collaborative care of patients with diabetes. Pharmacy Education. 2015; 15:1477-2701.
45. Park JJ, Kelly P, Carter BL, Burgess PP. Comprehensive pharmaceutical care in the chain setting. J Am Pharm Assoc. 1996; 36:443–451.

46. Paulo PT, Medeiros PA. A randomised clinical trial of the impact of pharmaceutical care on the health of type 2 diabetic patients. Lat Am J Pharm. 2016; 35: 1361-1368.
47. Paulos CP, Akesson Nygren CE, Celedon C, Carcamo CA. Impact of a pharmaceutical care program in a community pharmacy on patients with dyslipidemia. Ann Pharmacother. 2005; 39:939–943
48. Planas LG, Crosby KM, Mitchell KD, Farmer KC. Evaluation of a hypertension medication therapy management program in patients with diabetes. JAPhA. 2009; 49:164–170.
49. Plaster CP, Melo DT, Boldt V, Cassaro KOS, Lessa FCR, Boëchat GAP, Bissoli NS, de Andrade TU. Reduction of cardiovascular risk in patients with metabolic syndrome in a community health center after a pharmaceutical care program of pharmacotherapy follow-up. Braz J Pharm Sci. 2012; 48:435-446.
50. Polgreen LA, Han J, Carter BL, Ardery GP, Coffey CS, Chrischilles EA, James PA. Cost effectiveness of a physician-pharmacist collaboration intervention to improve blood pressure control. Hypertension. 2015; 66:1145–1151.
51. Robinson JD, Segal R, Lopez LM, Doty RE. Impact of a pharmaceutical care intervention on blood pressure control in a chain pharmacy practice. Ann Pharmacother. 2010; 44:88–96.
52. Rothman RL, Malone R, Bryant B, Shintani AK, Crigler B, Dewalt DA, Dittus RS, Weinberger M, Pignone MP. A randomized trial of a primary care-based disease management program to improve cardiovascular risk factors and glycated hemoglobin levels in patients with diabetes. Am J Med. 2005; 118:276–284.
53. Sanchez-Guerra J, Lopez y Lopez G, García-Jiménez S, Ávila-Jiménez L, Gómez-Galicia D, Carreras-Olivares B, Toledano-Jaimes C. Impact of a pilot program of medication review with follow-up on the blood pressure control in hypertension ambulatory patients with metabolic syndrome in Mexico. Pharm Care Esp. 2018; 20: 3-26
54. Scott DM, Boyd ST, Stephan M, Augustine SC, Reardon TP. Outcomes of pharmacist-managed diabetes care services in a community health center. Am J Health Syst Pharm. 2006; 63:2116–2122.
55. Shao H, Chen G, Zhu C, Chen Y, Liu Y, He Y, Jin H. Effect of pharmaceutical care on clinical outcomes of outpatients with type 2 diabetes mellitus. Patient Prefer Adher. 2017; 11:897–903.
56. Simpson SH, Majumdar SR, Tsuyuki RT, Lewanczuk RZ, Spooner R, Johnson JA. Effect of Adding Pharmacists to Primary Care Teams on Blood Pressure Control in Patients with Type 2 Diabetes: A randomized controlled trial. Diabetes Care. 2011; 34:20–26.
57. Skowron A, Polak S, Brandys J. The impact of pharmaceutical care on patients with hypertension and their pharmacists. Pharmacy Pract. 2011; 9:110-115.
58. Sookaneknun P, Richards RME, Sanguansermsri J, Teerasut C. Pharmacist involvement in primary care improves hypertensive patient clinical outcomes. Ann Pharmacother. 2004; 38:2023–2028.
59. Stewart K, George J, Mc Namara KP, Jackson SL, Peterson GM, Bereznicki LR, Gee PR, Hughes JD, Bailey MJ, Hsueh YA, McDowell JM, Bortoletto DA, Lau R. A multifaceted pharmacist intervention to improve antihypertensive adherence: a cluster-randomized, controlled trial (HAPPy trial). J Clin Pharm Ther. 2014; 39: 527–534.
60. Tahaineh L, Albsoul-Younes A, Al-Ashqar E, Habeb A. The role of clinical pharmacist on lipid control in dyslipidemic patients in North of Jordan. Int J Clin Pharm. 2011; 33: 229.

61. Taylor CT, Byrd DC, Krueger K. Improving primary care in rural Alabama with a pharmacy initiative. Am J Health Syst Pharm. 2003; 60:1123–1129.
62. Taylor SJ, Milanova T, Hourihan F, Krass I, Coleman C, Armour CL. A cost-effectiveness analysis of a community pharmacist-initiated disease state management service for type 2 diabetes mellitus. Int J Pharm Pract. 2005; 13: 33-40.
63. Tobari H, Arimoto T, Shimojo N, Yuhara K, Noda H, Yamagishi K, Iso H. Physician-pharmacist cooperation program for blood pressure control in patients with hypertension: a randomized-controlled trial. Am J Hypertens. 2010; 23:1144–1152.
64. Torres A, Fité B, Gascón P, Barau R, Guayta-Escolies M, Estrada-Campmany C, Rodríguez, C. Efectividad de un programa de atención farmacéutica en la mejora del control de la presión arterial en pacientes hipertensos mal controlados. Estudio PressFarm. Hipertens Riesgo Vasc. 2010; 27: 13–22.
65. Villa LA, Von Chrismar AM, Oyarzun C, Eujenin P, Fernandez ME, Quezada M. Pharmaceutical Care Program for dyslipidemic patients at three primary health care centers: impacts and outcomes. Latin Am J Pharm. 2009; 28:415-420.
66. Villeneuve J, Genest J, Blais L, Vanier MC, Lamarre D, Fredette M, Lussier MT, Perreault S, Hudon E, Berbiche D, Lalonde L. A cluster randomized controlled Trial to Evaluate an Ambulatory primary care Management program for patients with dyslipidemia: the TEAM study. CMAJ. 2010; 182:447–455.
67. Wal P, Wal A, Bhandari A, Pandey U, Rai AK. Pharmacist involvement in the patient care improves outcome in hypertension patients. J Res Pharm Pract. 2013; 2:123-129.
68. Wang J, Wu J, Yang J, Zhuang Y, Chen J, Qian W, Tian J, Chen X, She D, Peng F. Effects of pharmaceutical care interventions on blood pressure and medication adherence of patients with primary hypertension in China. Clin Res Regul Aff. 2010; 28:1-6
69. Wishah RA, Al-Khawaldeh OA, Albsoul AM. Impact of pharmaceutical care interventions on glycemic control and other health-related clinical outcomes in patients with type 2 diabetes: randomized controlled trial. Diabetes Metab Syndr. 2015; 9:271–276.
70. Zillich AJ, Sutherland JM, Kumbera PA. Hypertension outcomes through blood pressure monitoring and evaluation by pharmacists (HOME Study). J Gen Intern Med. 2005; 20:1091-1096.
